# Supplementary material for: Function and regulation of a steroidogenic CYP450 enzyme in the mitochondrion of Toxoplasma gondii
Source: PLoS Pathog. 2023 Aug 31;19(8):e1011566. doi: 10.1371/journal.ppat.1011566 (PMC10499268; doi:10.1371/journal.ppat.1011566)
Supplement: S1 Fig — (PDF) [file ppat.1011566.s001.pdf]

Regression Equations

No.	Name	Transition	RT (min)	Regression equation	R2
1	Aldosterone	361.3 -> 343.2	2.269	y = 40.941895 * x + 14.374474	0.99900918
2	Cortisone	361.3 -> 163.0	2.740	y = 49.407611 * x + 8.221463	0.99748228
3	Corticosterone	347.2 -> 329.2	4.025	y = 61.491594 * x + 6.005760	0.99854549
4	11-Deoxycortisol	347.1 -> 97.0	4.268	y = 117.075413 * x - 1.346635	0.99745509
5	19-Norandrostenedione	273.2 -> 109.0	6.647	y = 123.593646 * x + 18.329943	0.99846664
6	β- Nortestosterone	275.2 -> 109.1	5.419	y = 135.983148 * x + 14.973110	0.99925969
7	4-Androstene-3,17-dione	287.2 -> 97.1	7.316	y = 187.832766 * x - 2.299437	0.99942783
8	11-Deoxycorticosterone	331.3 -> 97.0	6.838	y = 226.464408 * x + 9.887778	0.99909757
9	2-Methoxyestradiol	303.3 -> 137.0	6.812	y = 23.434330 * x + 3.510264	0.99764188
10	17-OH pregnenolone	333.3 -> 315.0	6.695	y = 4.556913 * x - 2.184276	0.99180284
11	Dehydroepiandrosterone	289.3 ->253.2	6.957	y = 7.066597 * x - 8.343892	0.99692389
12	17α- hydroxypregnenolone	333.3 -> 97.0	7.379	y = 3.869586 * x + 5.032868	0.98566526
13	Androstanediol	293.2 -> 275.2	6.721	y = 0.242x + 6.3263	0.9964
14	trans-Androsterone	291.2 -> 255.2	7.597	y = 13.970860 * x + 5.837701	0.99334199
15	Dihydroteststerone	291.2 -> 255.1	7.811	y = 47.449230 * x - 4.983867	0.99750844
16	cis-Androsterone	291.1 -> 255.2	8.640	y = 11.273485 * x + 18.034894	0.99345635
17	Pregnenolone	317.2 -> 299.3	9.036	y = 9.339932 * x - 2.360588	0.99000224
18	Hydrocortisone	363.1 -> 121.0	2.654	y = 50.183534 * x + 10.463118	0.99888096
19	Estradiol	273.1 -> 107.0	6.077	y = 6.130219 * x + 4.862953	0.99531557
20	Estrone	271.1 -> 253.1	7.204	y = 82.428979 * x + 9.118667	0.99713023
21	Testosterone	289.1 -> 97.0	6.416	y = 303.959486 * x + 1.987892	0.99951246
22	Estriol	289.1 -> 253.1	2.250	y = 6.011454 * x + 10.348710	0.99469571
23	Progesterone	315.2 -> 97.0	9.194	y = 319.166537 * x + 42.120155	0.99877300

Steroids Analysis Results_Creative Proteomics					
Concentration (ng/g)	Sample 1		Sample 2		
Compound	test-1	test-2	test-1	test-2	
Estriol	ND	ND	ND	ND	
Aldosterone	0.016	0.016	ND	ND	
Hydrocortisone	ND	ND	ND	ND	
Cortisone	0.147	0.154	ND	ND	
Corticosterone	0.196	0.187	ND	ND	
11-Deoxycortisol	0.032	0.033	ND	ND	
B-Nortestosterone	ND	ND	ND	ND	
Estradiol	ND	ND	ND	ND	
Testosterone	ND	ND	ND	ND	
5a-Androstanediol	1.430	1.483	ND	ND	
19-Norandrostenedione	ND	ND	ND	ND	
17OH- pregnenolone	9.356	10.647	ND	ND	
2-methoxyestradiol	ND	ND	ND	ND	
11-Deoxycorticosterone	2.052	2.062	ND	ND	
Dehydroepiandrosterone	2.906	3.156	ND	ND	
Estrone	ND	ND	ND	ND	
4-Androstene-3,17-dione	0.800	0.839	ND	ND	
17a-hydroxypregnenolone	2.082	2.108	ND	ND	
trans-androsterone	0.657	0.755	ND	ND	
Dihydroteststerone	0.353	0.289	ND	ND	
cis-Androsterone	ND	ND	ND	ND	
pregnenolone	0.120	0.120	ND	ND	
Progesterone	0.481	0.499	ND	ND	
ND: not detected					

Figure S1. Analytical results of steroid detected in *Toxoplasma:* Regression Equations and Calculation Results (from Creative Proteomics)
