# Supplementary material for: Function and regulation of a steroidogenic CYP450 enzyme in the mitochondrion of Toxoplasma gondii
Source: PLoS Pathog. 2023 Aug 31;19(8):e1011566. doi: 10.1371/journal.ppat.1011566 (PMC10499268; doi:10.1371/journal.ppat.1011566)
Supplement: S2 Fig — (A) Sequence comparison between TgCYP450mt with closest human homologs that share the motif for heme-binding (red box). In blue are the six CYP450 localized to mitochondria. (B) C-terminal regions of CYP450 homologs in Sarcocystidae as shown for Sarcocystis neurona (SN3_00900360), Cystoisospora suis (CSUI_005351), Besnoitia besnoiti (BESB_078830), Neospora canimum (NCLIV_058440), Toxoplasma gondii (TGME49_315770) and Hammondia hammondi (HHA_315770). In green: helix K domain to stabilize the protein core and in yellow: heme-binding loop to position the iron atom in the heme. (PDF) [file ppat.1011566.s002.pdf]

A. Conserved residues in N-terminal TgCYP450

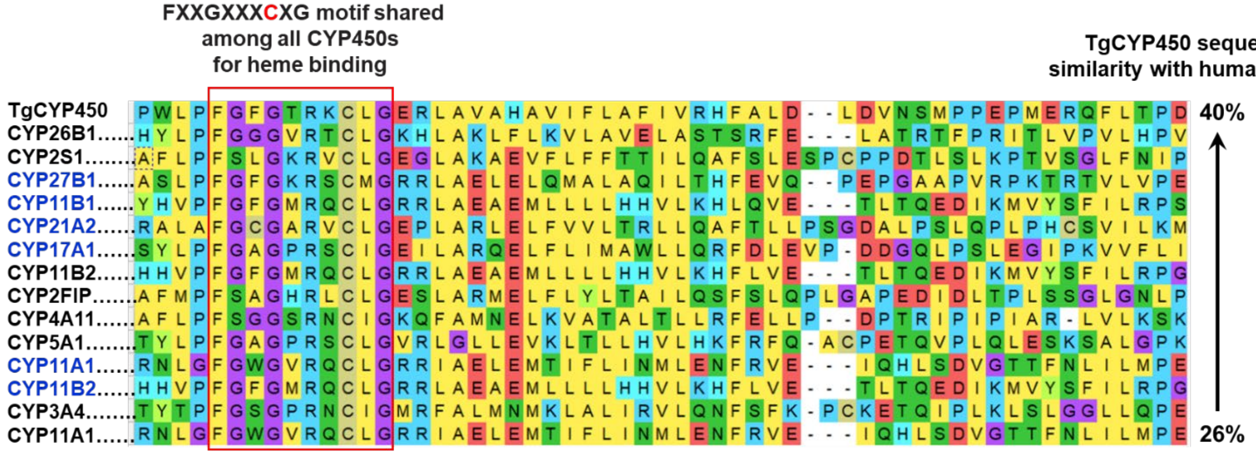

B. C-termini of CYP450 homologs in selected Sarcocystidae

```
Sarcocystis      FGEVMNLFLETLRFYPVPAITRTAATSIVLKAVNITIPCGSLVVVDNFSLTRDPVLWGAD      473
Cystoisospora    LGEVLNLFLETLRYYPVPVLSRTAAESIYIKSGDFTIPSGTVVLVDNYSLTRDRSVWGED      478
Besnoitia        LGEVLNLFLETLRFYALPALSRTASARIDLKSVDISIPAGTVIMVDNHSLTRDQARWGSD      466
Neospora         LGEVLNLFLEALRFYALPILSRSASSPIQLKSADLTIPAGTVVLVDNYSLTRDETLWGQD      412
Toxoplasma       LGEVVNVFLESLRFYALPILSRSASKAIHLKSVDFSIPAGTVILVDNYSLTRDEVLWGQD      470
Hammondia        LGEVLNVFLESLRFYALPILSRSASKAIHLKSVDFSIPAGTVILVDNYSLTRDEVLWGQD      470
                 :***:*:***:**:* :* ::*:*:  * :*: :::**.*::::***.*****   ** *

Sarcocystis      AEQFDPTRFVGRVWQQAPWVPFGFGPRKCLGPTSAITHAVVFLSFIIQHFSFSLDPHSVT      533
Cystoisospora    VDAFNPDRFIGRVWQHAPWIPFGFGSRKCLGERLAVTHAVFFLSFIVRHFILELDDNSMP      538
Besnoitia        AHLFNPDRFIGKIWQQAPWLPFGFGARKCLGERLSVTHAVLFLSSIVRSFALELDPNSMP      526
Neospora         ANVFNPDRFSGKIWQQAPWLPFGFGSRKCIGERVAVTHAVLFLAVVVRYFALELDTNSMP      472
Toxoplasma       ANVFNPDRFVGRIWQQAPWLPFGFGTRKCLGERLAVAHAVIFLAFIVRHFALDLDVNSMP      530
Hammondia        ANVFNPDRFVGRIWQQAPWLPFGFGARKCLGERLAVAHAVIFLAFIVRHFALDLDVNSMP      530
                 .. *:* ** *::**:***:***** ***:*   :::***.**: ::: * :.** :*:
```

Figure S2. Protein sequence alignment of CYP450 homologs

(A) Sequence comparison between TgCYP450mt with closest human homologs that share the motif for heme-binding (red box). In blue are the six CYP450 localized to mitochondria. (B) C-terminal regions of CYP450 homologs in Sarcocystidae as shown for *Sarcocystis neurona* (SN3_00900360), *Cystoisospora suis* (CSUI_005351), *Besnoitia besnoiti* (BESB_078830), *Neospora canimum* (NCLIV_058440), *Toxoplasma gondii* (TGME49_315770) and *Hammondia hammondi* (HHA_315770). In green: helix K domain to stabilize the protein core and in yellow: heme-binding loop to position the iron atom in the heme.
