# Supplementary material for: Function and regulation of a steroidogenic CYP450 enzyme in the mitochondrion of Toxoplasma gondii
Source: PLoS Pathog. 2023 Aug 31;19(8):e1011566. doi: 10.1371/journal.ppat.1011566 (PMC10499268; doi:10.1371/journal.ppat.1011566)
Supplement: S3 Fig — See detailed description of molecular constructs in the Materials and Methods section. (A) Strategy for in-situ C-terminal tagging with 3xHA with the red region denoting the stop codon and the dotted black line the CRISPR/Cas9- mediated cut, verified by PCR and Western blotting using an anti-HA antibody and identifying 7 positive clones (shown in the lanes). (B) Promotor displacement strategy for conditional expression of the TgCYP450mt gene under the control of tetracycline-dependent SAG4 promotor, confirmed by PCR and Western blotting using anti-HA antibody showing down-expression of TgCYP450mt-HA after exposure to 1μg/ml of anhydrous tetracycline for 24 h (~70% decrease) and 48 h (~90% decrease), compared to no added tetracycline. Loading control anti-TgTubulin antibody was used to assess protein expression levels and validate Western blot analysis. (C) Strategy for direct gene deletion of TgMAPR via double homologous recombination as confirmed by PCR and Western blotting using anti-TgMAPR antibody. (D) Strategy for introduction of the TgMAPR gene on ΔTgMAPR parasites. Successful gene complementation was probed by PCR, Western blotting using anti-HA antibody and IFA using two different anti-HA antibodies. (PDF) [file ppat.1011566.s003.pdf]

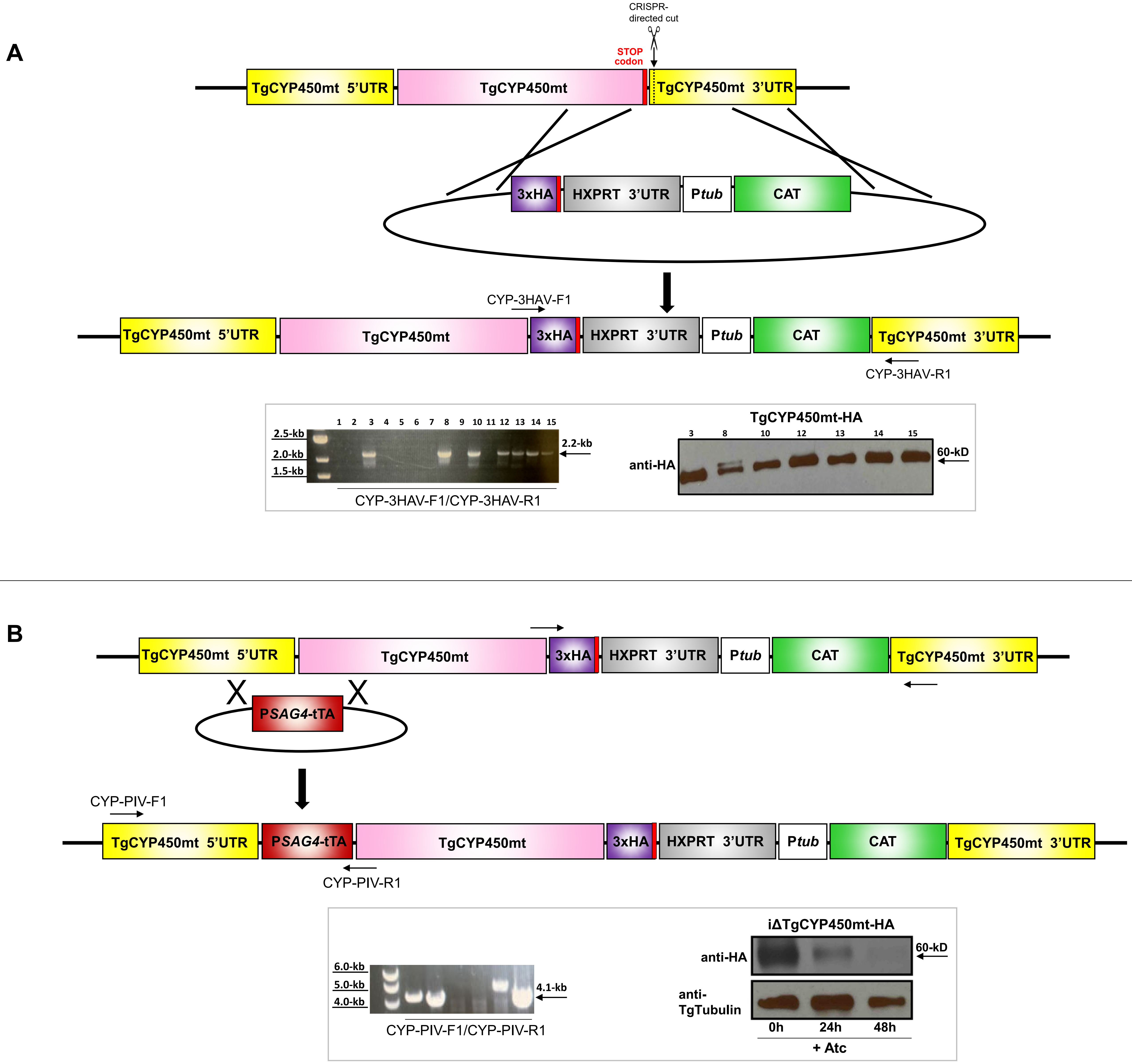
A
CRISPR-
directed cut
STOP
codon
TgCYP450mt 5'UTR
TgCYP450mt
TgCYP450mt 3'UTR
3xHA
HXPRT 3'UTR
Ptub
CAT
CYP-3HAV-F1
CYP-3HAV-R1
1 2 3 4 5 6 7 8 9 10 11 12 13 14 15
2.5-kb
2.0-kb
1.5-kb
2.2-kb
CYP-3HAV-F1/CYP-3HAV-R1
TgCYP450mt-HA
3 8 10 12 13 14 15
anti-HA
60-kD
B
PSAG4-tTA
CYP-PIV-F1
CYP-PIV-R1
6.0-kb
5.0-kb
4.0-kb
4.1-kb
CYP-PIV-F1/CYP-PIV-R1
iΔTgCYP450mt-HA
anti-HA
60-kD
anti-
TgTubulin
0h
24h
48h
+ Atc

(to be continued)

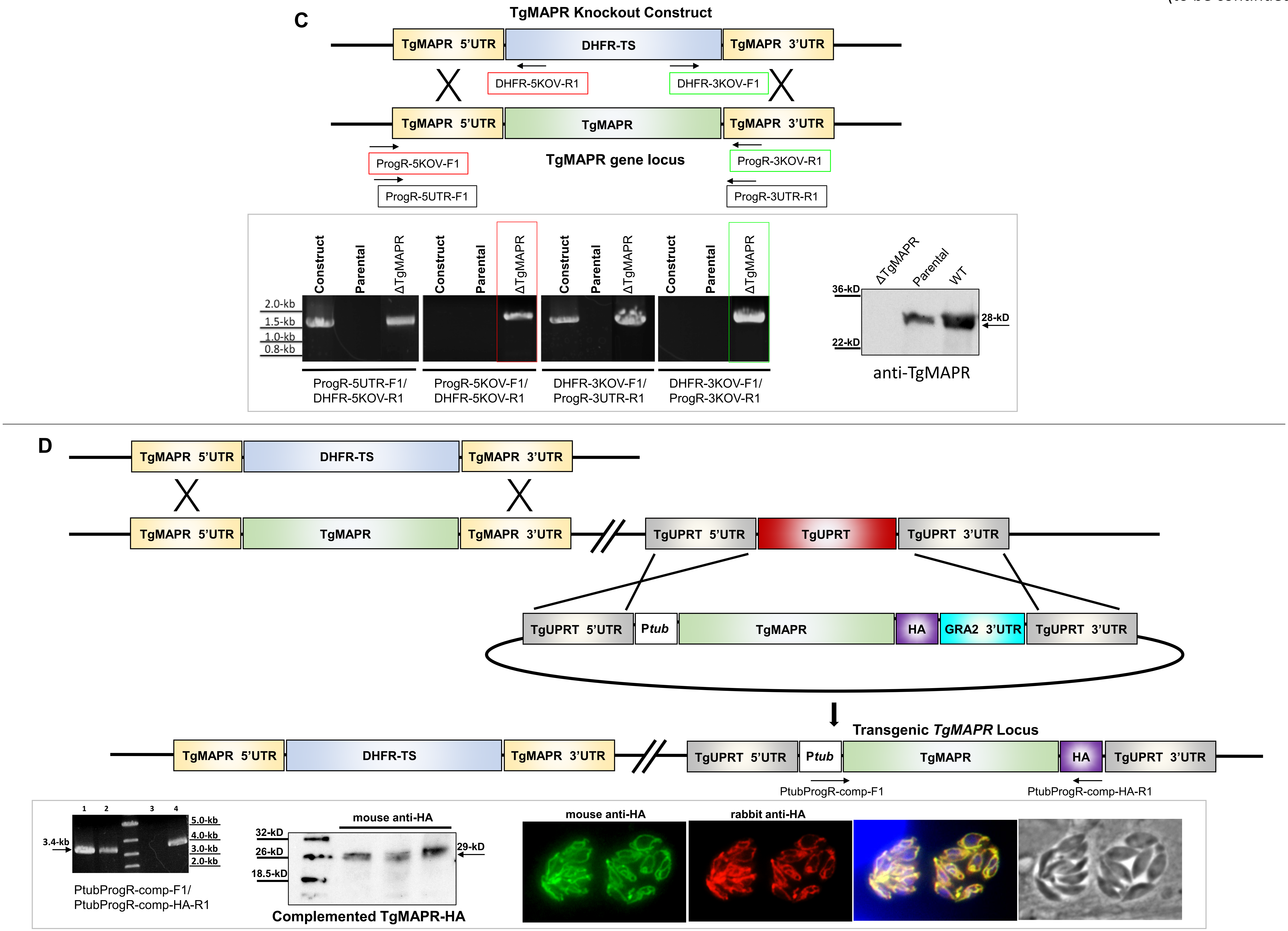

Figure S3. Schemas describing the strategies to generate the parasite strains used in this study

See detailed description of molecular constructs in the Materials and Methods section.

(A) Strategy for in-situ C-terminal tagging with 3xHA with the red region denoting the stop codon and the dotted black line the CRISPR/Cas9- mediated cut, verified by PCR and Western blotting using an anti-HA antibody and identifying 7 positive clones (shown in the lanes). (B) Promotor displacement strategy for conditional expression of the *TgCYP450mt* gene under the control of tetracycline-dependent SAG4 promotor, confirmed by PCR and Western blotting using anti-HA antibody showing down-expression of TgCYP450mt-HA after exposure to 1µg/ml of anhydrous tetracycline for 24 h (~70% decrease) and 48 h (~90% decrease), compared to no added tetracycline. Loading control anti-TgTubulin antibody was used to assess protein expression levels and validate Western blot analysis. (C) Strategy for direct gene deletion of TgMAPR via double homologous recombination as confirmed by PCR and Western blotting using anti-TgMAPR antibody. (D) Strategy for introduction of the TgMAPR gene on ΔTgMAPR parasites. Successful gene complementation was probed by PCR, Western blotting using anti-HA antibody and IFA using two different anti-HA antibodies.
