# Supplementary material for: Function and regulation of a steroidogenic CYP450 enzyme in the mitochondrion of Toxoplasma gondii
Source: PLoS Pathog. 2023 Aug 31;19(8):e1011566. doi: 10.1371/journal.ppat.1011566 (PMC10499268; doi:10.1371/journal.ppat.1011566)
Supplement: S4 Fig — (A) In Sarcocystidae, shown for Neospora caninum (NCLIV_060960), Besnoitia besnoiti (BESB_027250), Toxoplasma gondii (TGME49_276990; TgMAPR) and Hammondia hammondi (HHA_276990). Shown are N-terminal sequences. Highlighted in green: one predicted transmembrane domain and highlighted in turquoise: the MAPR/Cytb5 domain with the sole axial tyrosinate residue (blue box) for the iron chelation of heme. (B) Sequence comparison between TgMAPR, human PGRMC1 (HsPGRMC1; Swiss-Prot O00264) and Schizosaccharomyces pombe (SpDap1; SPAC25B8.01) that share the MAPR/Cytb5 domain (highlighted in turquoise); the conserved tyrosine and aspartic acid residues required for heme binding (blue boxes); potential phosphorylation sites (green boxes); potential SH2 domain (green boxes at the C-terminus) and SH3 domain (fuchsia box). The three residues in yellow typify the PGRMC/Dap1 proteins, with two present in TgMAPR. (PDF) [file ppat.1011566.s004.pdf]

A. N-terminus of MAPR homologs in selected Sarcocystidae

```
Neospora        MLLTTVKQSLEKLDFWTVAGIVLGSLATYKMIAS--LCHRSAGSSSRGDSAGDGASHAAR 58
Besnoitia       -----MKEWLQKLDFWTVAGVAVGSFAAYKMLSASLGYFSSSASSARLGGSSGGRQTCNE 55
Toxoplasma      MVLTMMKQWVEKLDFWTIAGIVLGSLATYKMVKASLRCHSFWGNPA-----NDGTRQAAS 55
Hammondia       MLLTMMKQWVEKLDFWTIAGIVLGSLATYKMVKASLRCHSFWGNPA-----SDGTRQATS 55
                     :*: ::******:**:.:**:*:***: :    .   .. :     ..*   .

Neospora        NAGGGYKAPPKPNPCPRDFTLKELCQFNGNQKPPVAKMA-EAPASEASAGGHTSEPRDAA 117
Besnoitia       GEDEYYRAPPKPKPCPRDFTLEELRPFDGTQTAPAGLEAPAAAPGEGDAGVHPPEDLDPA 115
Toxoplasma      SADSAYKAPPKPNPCPRDFTLDELRAFNGNQKPPVAKAT-EERVAEVSSGVRTPEAMDAA 114
Hammondia       SADRAYKAPPKPNPCPRDFTLDELRGFNGNQKPLVAKAT-EAPAAEASSGVRTPEAMDAA 114
                . .  *:*****:********.**  *:*.*.  ..  :     .*  .:* :  *  * *

Neospora        SVSATAAAAVGALPPIYLALKGRVYDVTSHRDGRRFYAPDGPYGVFAGSDVTMNLAKMVF 177
Besnoitia       SASAVAAAAVGALPPIYIALKGRVYDVTSHPDGRHFYSADGPYGIFAGKDVTVNLAKMVF 175
Toxoplasma      SASAVAAAAVGASPPIYIALKGRVYDVTSHRDGRRFYGADGPYGIFAGSDVTMNLAKMVF 174
Hammondia       SASAVAAAAVGALPPIYLALKGRVYDVTSHRDGRRFYAADGPYGIFAGSDVTMNLAKMVF 174
                *.**.******* ****:************ ***:**. *****:***.***:*******

Neospora        EESEKNQVRAIKVADSFLSRA-------------ADRGRLGGALPSQIRSCRICGVWSAI 224
Besnoitia       DESENNQGPSAWATLSALEKQTVDDWEDRFRAKYAPVGYVVFGHPEE--DAKIREIY--- 230
Toxoplasma      SEGEKNTVPSKWQTLSPDEKETIDDWEERFKAKYDHVGFVVFGVPAE--DALLREMY--- 229
Hammondia       SEGEKNTVPSKWQTLSPAEKETVEDWEERFKAKYDHVGFVVFGVPAE--DALLREMY--- 229
                .*.*:*   :   : *  .:                 * :  . * :  .. :  ::
```

B. Conserved residues in TgMAPR

```
HsPGRMC1        -MAAEDVVATGADPSDLESGGLLHEIFTSPLNLLLLGLCIFLLYKIVRGDQPAASGDSDD
SpDap1          ---------------------------MASTQVVFIVTLFLYLLITRWRRKNEKSFIASEE
TgMAPR          MVLTMMKQWVEKLDFWTIAGIVLGSLATYKMVKASLRCHSFWGNPANDGTRQAASSADSA
                                           :       :    :         .  :   ..

HsPGRMC1        DEPPPLPRIKRRDFTPAELRRFDGVQ----------------------------------
SpDap1          PKQP-----EWRDYTPAELKEYNGSK----------------------------------
TgMAPR          YKAPPKPNPCPRDFTLDELRAFNGNQKPPVAKATEERVAEVSSGVRTPEAMDAASASAVA
                 : *       **:*  **: ::* :

HsPGRMC1        ------DPRILMAINGKVFDVT---KGRKFYGPEGPYGVFAGRDASRGLATFCLDKEALK
SpDap1          ------NSLVFLAIKGTVYNVT---MGSKFYGPQGPYSAFAGHDASRGLAKNSFDDEFIP
TgMAPR          AAAVGASPPIYIALKGRVYDVTSHRDGRRFYGADGPYGIFAGSDVTMNLAKMVFSEGEKN
                      .. : :*::* *::**    * :***.:***. *** *.: .**.  :..

HsPGRMC1        D----EYDDLSDLTAAQQETLSDWESQFTFKYHHVGKLLK--EGEEPTVYSDEEEPKDES
SpDap1          DSDAEELDDCSDLNDEERQALNDWKAFFDQKYQAVGRLIS--PREARAAATISETEEKVA
TgMAPR          TVP----SKWQTLSPDEKETIDDWEERFKAKYDHVGFVVFGVPAEDALLREMYSEERKEA
                       .. . *.  :::::.**:  *  **. ** ::     *        .  .. :

HsPGRMC1        ARKND
SpDap1          HN---
TgMAPR          GLAV-
```

Figure S4. Protein sequence alignment of MAPR homologs

(A) In Sarcocystidae, shown for *Neospora caninum* (NCLIV_060960), *Besnoitia besnoiti* (BESB_027250), *Toxoplasma gondii* (TGGT1_276990; TgMAPR) and *Hammondia hammondi* (HHA_276990). Shown are N-terminal sequences. Highlighted in green: one predicted transmembrane domain and highlighted in turquoise: the MAPR/Cytb5 domain with the sole axial tyrosinate residue (blue box) for the iron chelation

of heme. (B) Sequence comparison between TgMAPR, human PGRMC1 (HsPGRMC1; Swiss-Prot O00264) and *Schizosaccharomyces pombe* (SpDap1; SPAC25B8.01) that share the MAPR/Cytb5 domain (highlighted in turquoise); the conserved tyrosine and aspartic acid residues required for heme binding (blue boxes); potential phosphorylation sites (green boxes; residues in brown); potential SH2 domain (green boxes at the C-terminus) and SH3 domain (fuchsia box). The three residues in yellow typify the PGRMC/Dap1 proteins, with two present in TgMAPR.
