# Supplementary material for: Function and regulation of a steroidogenic CYP450 enzyme in the mitochondrion of Toxoplasma gondii
Source: PLoS Pathog. 2023 Aug 31;19(8):e1011566. doi: 10.1371/journal.ppat.1011566 (PMC10499268; doi:10.1371/journal.ppat.1011566)
Supplement: S5 Fig — (A) Immunofluorescence for localization of TgMAPR in mitochondria in HeLa cells transfected with TgMAPR-HA; anti-HA (green), Mito-T (red). (B) Double IFA on TgMAPR-HA-expressing Toxoplasma using anti-HA and anti-TgMAPR antibodies showing colocalization with positive PPC. (C) Western blots on freeze-thaw lysates from TgMAPR-HA-expressing Toxoplasma using anti-TgHA antibody revealing 2 bands corresponding to monomeric and dimeric forms of TgMAPR-HA. (D) ImmunoEM on TgMAPR-HA-expressing Toxoplasma using anti-HA antibody confirming gold particles on mitochondrial membranes (white arrow on the outer membrane and black arrow on the inner membrane). (E-F) Double IFA on TgMAPR-HA-expressing Toxoplasma using anti-HA and anti-HSP70 antibodies showing colocalization. Values of PCC and MOC are shown. Arrows in F show area enriched in TgMAPR. (PDF) [file ppat.1011566.s005.pdf]

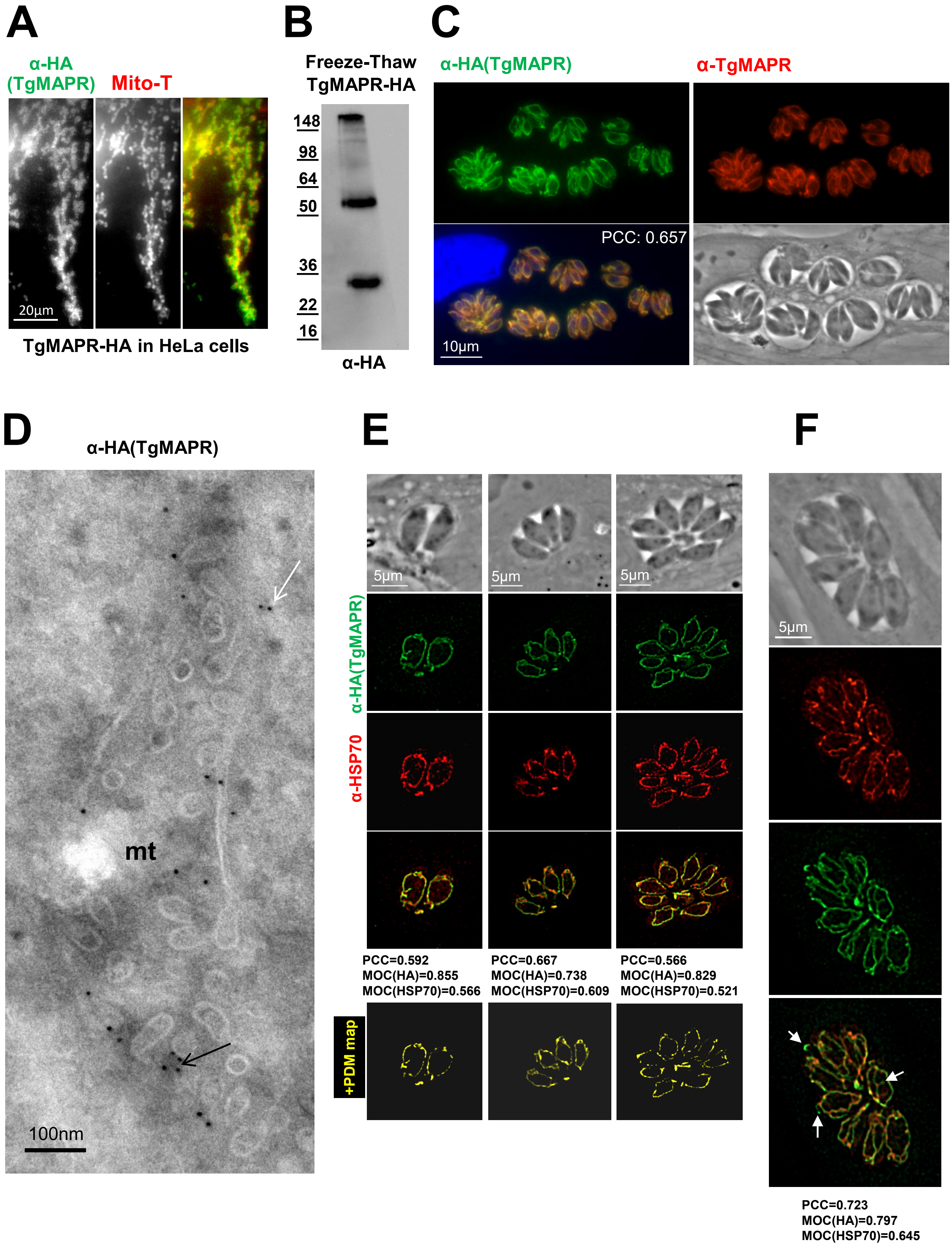

Figure S5. TgMARP localization in mammalian cells and transgenic parasites

Figure S5. Mitochondrial localization of TgMAPR

(A) Immunofluorescence for localization of TgMAPR in mitochondria in HeLa cells transfected with TgMAPR-HA; anti-HA (green), Mito-T (red). (B) Double IFA on TgMAPR-HA-expressing *Toxoplasma* using anti-HA and anti-TgMAPR antibodies showing colocalization with positive PPC. (C) Western blots on freeze-thaw lysates from TgMAPR-HA-expressing Toxoplasma using anti-TgHA antibody revealing 2 bands corresponding to monomeric and dimeric forms of TgMAPR-HA. (D) ImmunoEM on TgMAPR-HA-expressing *Toxoplasma* using anti-HA antibody confirming gold particles on mitochondrial membranes (white arrow on the outer membrane and black arrow on the inner membrane. (E-F) Double IFA on TgMAPR-HA-expressing *Toxoplasma* using anti-HA and anti-HSP70 antibodies showing colocalization. Values of PCC and MOC are shown. Arrows in F show area enriched in TgMAPR.
