# Supplementary material for: Function and regulation of a steroidogenic CYP450 enzyme in the mitochondrion of Toxoplasma gondii
Source: PLoS Pathog. 2023 Aug 31;19(8):e1011566. doi: 10.1371/journal.ppat.1011566 (PMC10499268; doi:10.1371/journal.ppat.1011566)
Supplement: S6 Fig — (A) Expression of TgMAPR in yeast SpDap1Δ under hypoxic conditions. Panel i: Western blots of TgMAPR-myc-expressing Dap1Δ of S. pombe using anti-myc antibody, verifying TgMAPR expression, and 3xHA-SpDAP1 expressed in Dap1Δ of S. pombe using anti-HA antibody, verifying the complementation. Panel ii: Yeast growth assay at 30°C for 3 days in medium containing CoCl2, showing rescue of growth of dap1Δ upon expression of either SpDap1 or TgMAPR, with comparable growth to the yeast parental strain (panel b). (B) Sterol profiles in yeast SpDap1Δ expressing TgMAPR. Ergosterol biosynthetic pathway in yeast showing the role for Dap1p in activating Erg11p is shown in the inset. Sterols extracted from log-phase WT, parental, Spdap1Δ yeast expressing Dap1 (positive control, vector alone (negative control) or TgMAPR were analyzed by gas chromatography and GC-MS. Data are the average of three independent replicates ± SD. (PDF) [file ppat.1011566.s006.pdf]

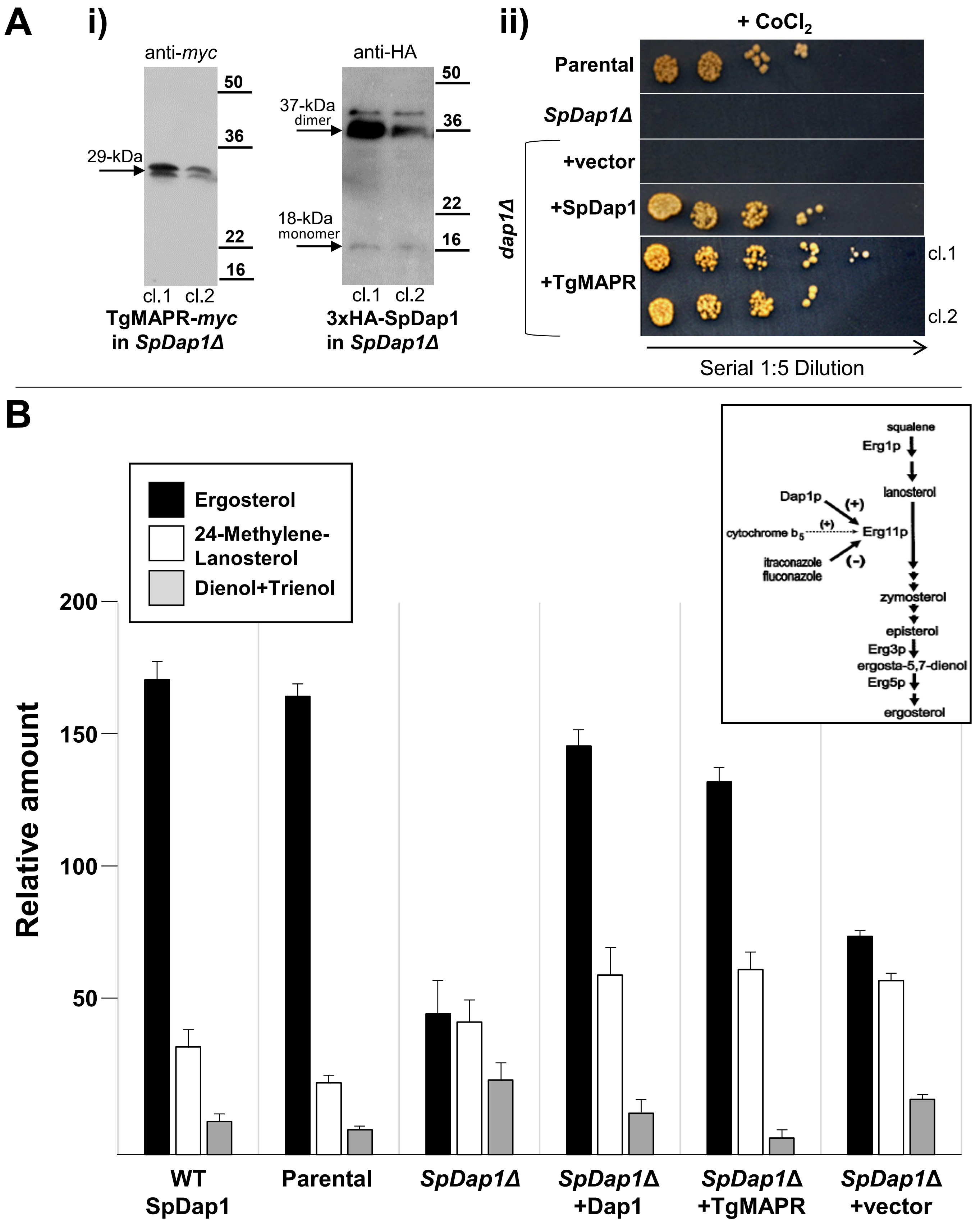

Figure S6. Functional complementation of yeast Dap1Δ by TgMAPR

Figure S6. Functional complementation of SpDap1Δ with TgMAPR

(A) Expression of TgMAPR in yeast *SpDap1Δ* under hypoxic conditions**.** Panel i: Western blots of TgMAPR-*myc*-expressing *Dap1Δ* of *S. pombe* using anti-myc antibody, verifying TgMAPR expression. 3xHA-SpDAP1 expressed in Dap1Δ of S. pombe using anti-HA antibody, verifying the complementation. Panel ii: Yeast growth assay at 30°C for 3 days in medium containing $CoCl_2$, showing rescue of growth of dap1Δ upon expression of either SpDap1 or TgMAPR, with comparable growth to the yeast parental strain (panel b). (B) Sterol profiles in yeast *SpDap1Δ* expressing TgMAPR. Ergosterol biosynthetic pathway in yeast showing the role for Dap1p in activating Erg11p is shown in the inset. Sterols extracted from log-phase WT, parental, *Spdap1Δ* yeast expressing Dap1 (positive control, vector alone (negative control) or TgMAPR were analyzed by gas chromatography and GC-MS. Data are the average of three independent replicates ± SD.
