# Supplementary material for: Function and regulation of a steroidogenic CYP450 enzyme in the mitochondrion of Toxoplasma gondii
Source: PLoS Pathog. 2023 Aug 31;19(8):e1011566. doi: 10.1371/journal.ppat.1011566 (PMC10499268; doi:10.1371/journal.ppat.1011566)
Supplement: S7 Fig — Purified parasites were labeled with propidium iodide to analyze cell cycle based on DNA content for experiments shown in Figs 4C(A) and 5E(B). (PDF) [file ppat.1011566.s007.pdf]

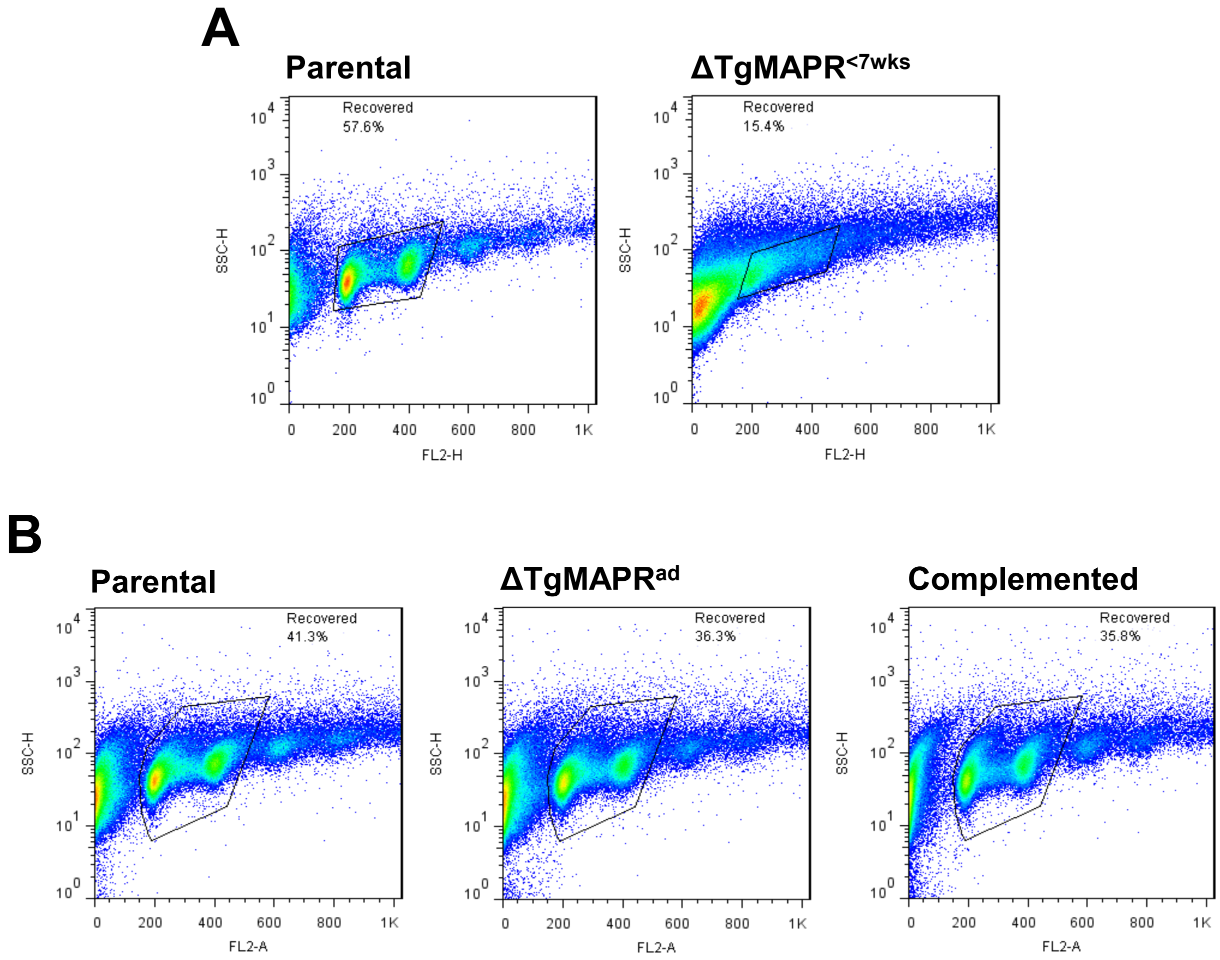

Figure S7. Gating strategy to select parasite populations based on side-scatter pulse height on the FL2 total area channel

Purified parasites were labeled with propidium iodide to analyze cell cycle based on DNA content for experiments shown in Figure 4C (A) and Figure 5E (B).
