# Supplementary material for: Function and regulation of a steroidogenic CYP450 enzyme in the mitochondrion of Toxoplasma gondii
Source: PLoS Pathog. 2023 Aug 31;19(8):e1011566. doi: 10.1371/journal.ppat.1011566 (PMC10499268; doi:10.1371/journal.ppat.1011566)
Supplement: S8 Fig — Volcano plot revealing 11 genes having increased expression (red) and 7 with decreased expression (green) in ΔTgMAPRad, with statistical significance less than 0.05, relative to ΔTgMAPR<7wks. The green and red dashed lines represent the borderline of Log2 fold change of 0.5 in gene transcripts, and the genes above the black dashed line had padj values of statistical significance below 0.05. Each sample was sequenced in duplicate for statistical comparison. Shown is the list of the 18 genes that are differentially expressed, with some identified in SWISS-PROT and ToxoDB. (PDF) [file ppat.1011566.s008.pdf]

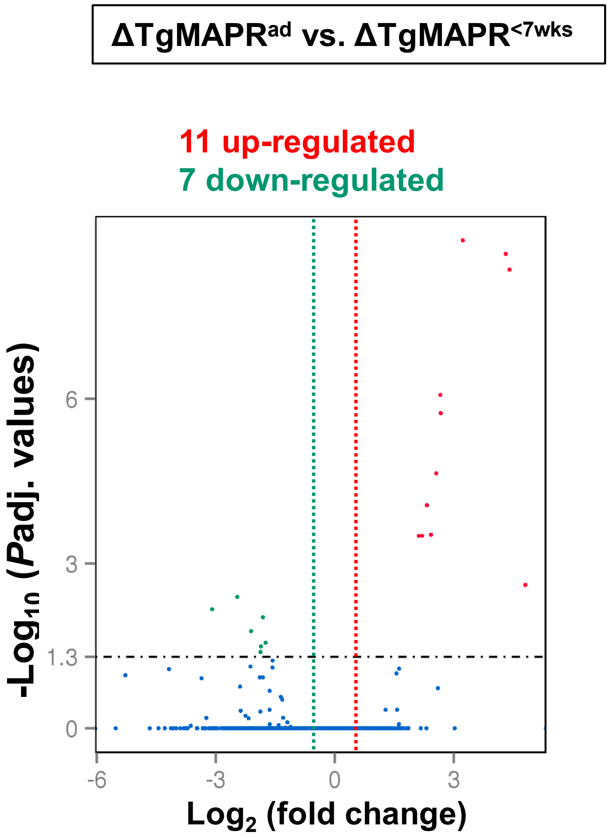

Gene_id	log2FoldChange	pval	padj	Blast swiss prot	ToxoDB
TGGT1_210478-t26_1	-3.0899	1.04E-05	0.0068046	-//-	hypothetical
TGGT1_240880-t26_1	-2.4575	5.69E-06	0.0040531	-//-	hypothetical
TGGT1_286932-t26_1	-2.1112	3.00E-05	0.017098	DYH6_HUMAN Dynein heavy chain 6	ATPase family associated with various cellular activities (AAA) domain-containing protein
TGGT1_275610-t26_1	-1.8743	8.62E-05	0.040929	CAMK1_ARATH CDPK-related kinase 1	Protein kinase
TGGT1_306338B-t26_1	-1.8616	6.41E-05	0.032212	DYHG_CHLRE Dynein gamma chain	putative dynein gamma chain
TGGT1_253930-t26_1	-1.8094	1.57E-05	0.0095545	SCUB2_DANRE Signal peptide	GCC2 and GCC3 domain-containing protein
TGGT1_212770-t26_1	-1.7439	5.18E-05	0.027644	-//-	hypothetical
TGGT1_293480-t26_1	2.1223	3.37E-07	0.00031335	CNX1_ARATH Molybdopterin biosynthesis protein	MoeA N-terminal region (domain I and II) domain-containing protein
TGGT1_310520-t26_1	2.1995	3.67E-07	0.00031335	sp|O60658|PDE8A_HUMAN High affinity cAMP-specific	3'5'-cyclic nucleotide phosphodiesterase domain-containing protein
TGGT1_211270-t26_1	2.3232	7.13E-08	8.71E-05	-//-	sushi domain (scr repeat) domain-containing protein
TGGT1_270580-t26_1	2.4227	2.80E-07	0.00029871	HERC1_HUMAN Probable E3 ubiquitin-protein ligase	HECT-domain (ubiquitin-transferase) domain-containing protein
TGGT1_226755-t26_1	2.5606	1.60E-08	2.27E-05	PDE3B_MOUSE cGMP-inhibited	3'5'-cyclic nucleotide phosphodiesterase domain-containing protein
TGGT1_237160-t26_1	2.6655	4.00E-10	8.55E-07	-//-	hypothetical
TGGT1_239020-t26_1	2.6703	1.07E-09	1.83E-06	MDR1_HUMAN Multidrug resistance protein 1	ABC transporter transmembrane region domain-containing protein
TGGT1_223060-t26_1	3.2253	1.53E-13	1.31E-09	MORN3_MACFA MORN repeat-containing protein	MORN repeat-containing protein
TGGT1_249840-t26_1	4.3103	5.42E-13	2.32E-09	DYH1_HUMAN Dynein heavy chain 1	putative dynein heavy chain 2
TGGT1_297840-t26_1	4.4086	1.57E-12	4.46E-09	PRI2_RAT DNA primase large subunit	DNA primase, large subunit
TGGT1_259115-t26_1	4.8054	3.15E-06	0.0024488	YP109_YEAST ABC1 family protein YPL109C, mitochondrial	ABC1 family protein

Figure S8. RNA-Seq comparison between ΔTgMAPR^{ad} and ΔTgMAPR$^{<7wks}$ parasites

Volcano plot revealing 11 genes having increased expression (red) and 7 with decreased expression (green) in ΔTgMAPR^{ad}, with statistical significance less than 0.05, relative to ΔTgMAPR$^{<7wks}$. The green and red dashed lines represent the borderline of Log2 fold change of 0.5 in gene transcripts, and the genes above the black dashed line had padj values of statistical significance below 0.05. Each sample was sequenced in duplicate for statistical comparison. Shown is the list of the 18 genes that are differentially expressed, with some identified in SWISS-PROT and ToxoDB.
