# Supplementary material for: Function and regulation of a steroidogenic CYP450 enzyme in the mitochondrion of Toxoplasma gondii
Source: PLoS Pathog. 2023 Aug 31;19(8):e1011566. doi: 10.1371/journal.ppat.1011566 (PMC10499268; doi:10.1371/journal.ppat.1011566)
Supplement: S9 Fig — Top. In situ interaction of TgCYP450mt and TgMAPR showing red fluorescent signal in PLA (see Fig 8B). Fluorescence microscopy of TgMAPR-HA- or TgCYP450-HA-expressing Toxoplasma using a PLA showing no fluorescent signal using primary antibody alone (anti-HA for each strain or anti-HSP70) or both primary antibodies (anti-HA for each strain and anti-HSP70). Nuclei staining by DAPI. (PDF) [file ppat.1011566.s009.pdf]

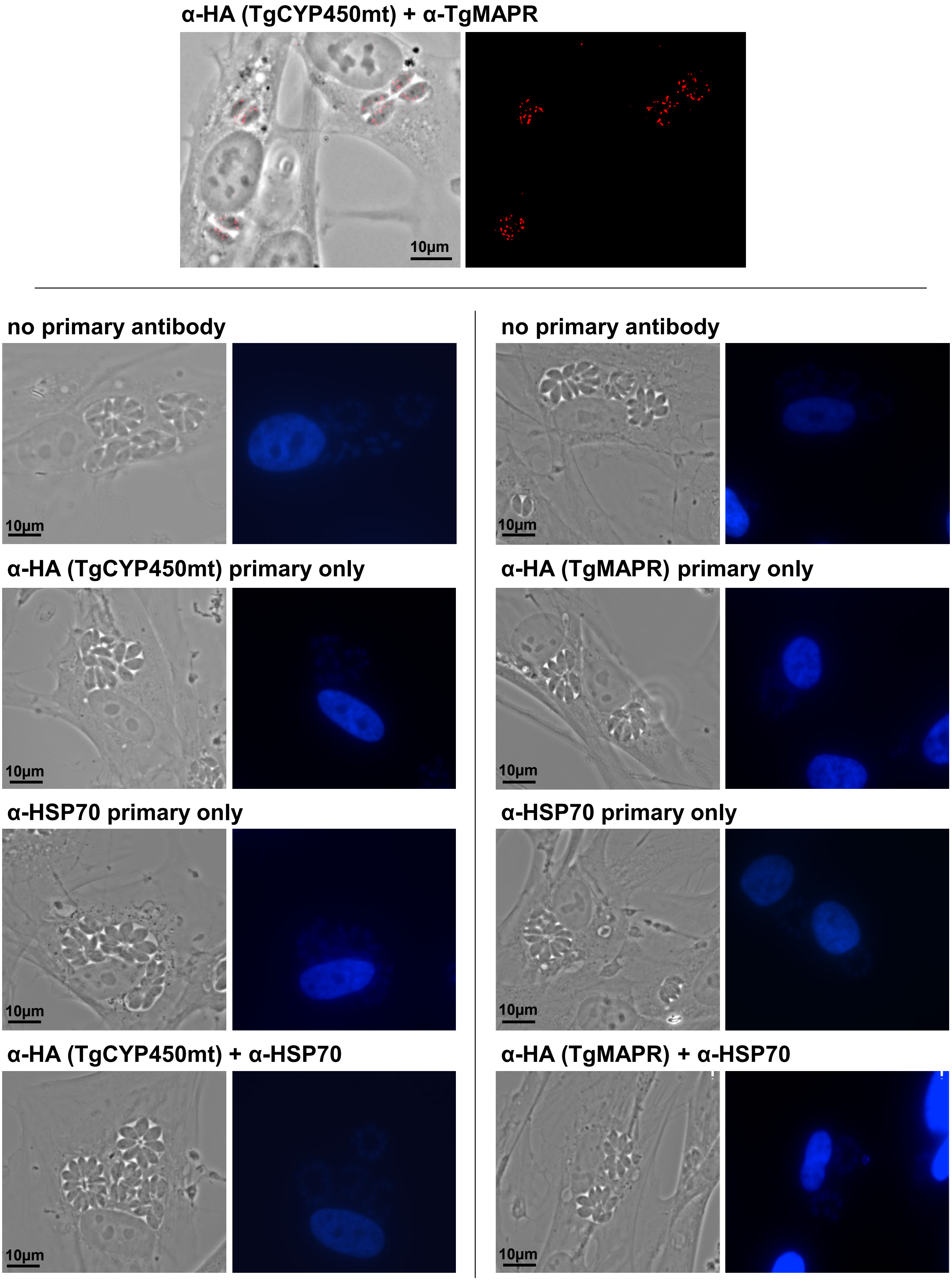

Figure S9. PLA control for TgCYP450mt and TgMAPR using anti-HSP70 antibody

Top. *In situ* interaction of TgCYP450mt and TgMAPR showing red fluorescent signal in PLA (see Fig. 8B). Fluorescence microscopy of TgMAPR-HA- or TgCYP450-HA-expressing *Toxoplasma* using a PLA showing no fluorescent signal using primary antibody alone (anti-HA for each strain or anti-HSP70) or both primary antibodies (anti-HA for each strain and anti-HSP70). Nuclei staining by DAPI.
