# Supplementary material for: Function and regulation of a steroidogenic CYP450 enzyme in the mitochondrion of Toxoplasma gondii
Source: PLoS Pathog. 2023 Aug 31;19(8):e1011566. doi: 10.1371/journal.ppat.1011566 (PMC10499268; doi:10.1371/journal.ppat.1011566)
Supplement: S10 Fig — In blue: mitochondrial enzymes with functions compatible to TgCYP450mt. (PDF) [file ppat.1011566.s010.pdf]

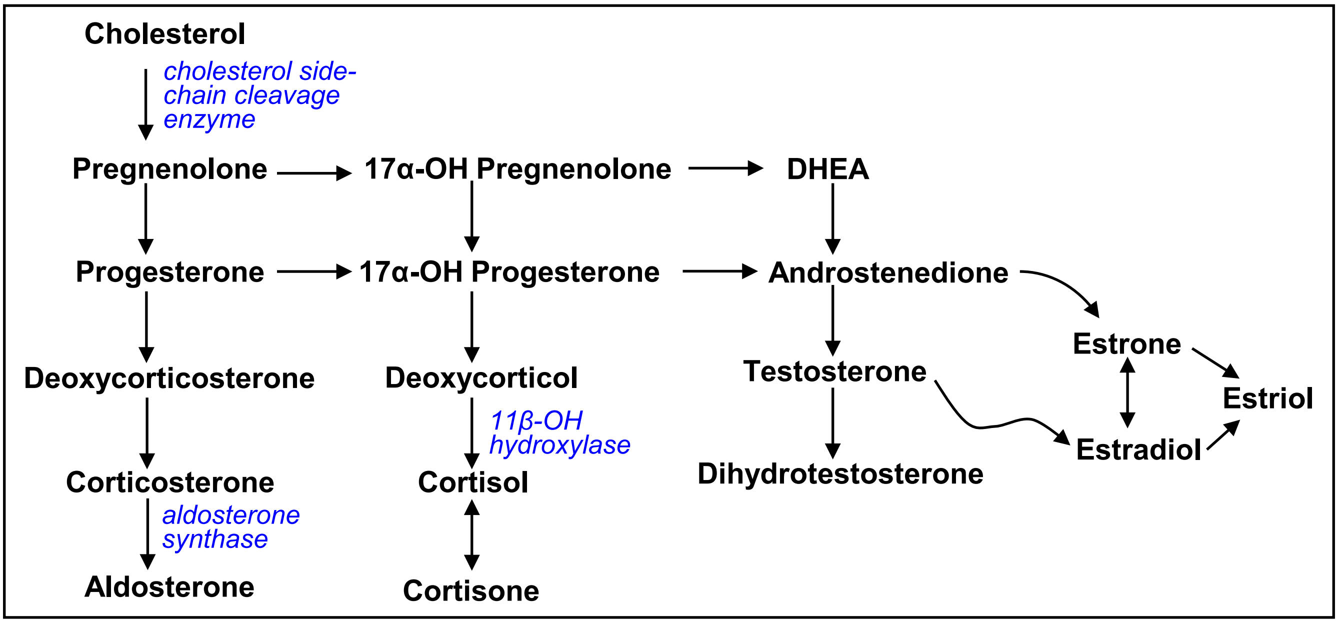

Figure S10. Schematic representation of steroid biosynthesis from cholesterol in Vertebrates.

In blue: mitochondrial enzymes with functions compatible to TgCYP450mt.
