# Supplementary material for: Function and regulation of a steroidogenic CYP450 enzyme in the mitochondrion of Toxoplasma gondii
Source: PLoS Pathog. 2023 Aug 31;19(8):e1011566. doi: 10.1371/journal.ppat.1011566 (PMC10499268; doi:10.1371/journal.ppat.1011566)
Supplement: S1 Table — (PDF) [file ppat.1011566.s011.pdf]

| **Primer Name** *(restriction site)* | **Primer Sequence** *(restriction sites underlined)* |
|---|---|
| **TgMAPR Localization of in *T. gondii*** | |
| ProgR-F1 *(BglII)* | ATCagatctATGGTGCTGACGATGATGAAGCAGTGGGTA |
| ProgR-HA-R1 *(AvrII)* | ATCcctaggttaagcgtaatctggaacatcgtatgggtaTACTGCAAGCCCTGCTTCCTTCCTCTCTTC |
| **Heterologous expression of TgMAPR in mammalian cells** | |
| ProgR-F2 *(EcoRI)* | ATCgaattcATGGTGCTGACGATGATGAAGCAGTGGGTA |
| ProgR-HA-R2 *(HindIII)* | ACTaagcttttaagcgtaatctggaacatcgtatgggtaTACTGCAAGCCCTGCTTCCTTCCTCTCTTC |
| **Heterologous expression of TgMAPR in *S. pombe*** | |
| ProgR-F3 *(BamHI)* | ATCggatccATGGTGCTGACGATGATGAAGCAGTGGGTA |
| ProgR-myc-R3 *(PstI)* | ACTctgcagTTAgaggtcttcttcggaaatcaacttctgttcTACTGCAAGCCCTGCTTCCTTCCTCTCTTC |
| **Recombinant expression of 6xHis-TgMAPR in *E. coli*** | |
| ProgR-F3 *(BamHI)* | ATCggatccATGGTGCTGACGATGATGAAGCAGTGGGTA |
| His-ProgR-R5 *(HindIII)* | ACTaagcttTTATACTGCAAGCCCTGCTTCCTTCCTCTC |
| **Deletion of TgMAPR** | |
| ProgR-5UTR-F1 (*ApaI*) | ATCgggcccTTTTACCCCACGAGGCAGGTGGCACTTCTT |
| ProgR-5UTR-R1 (*NheI*) | ATCgctagcGCTTAGGCAGGGTGGACACGTGTGAAGCGT |
| ProgR-3UTR-F1 (*SpeI*) | ATCactagtAGACGCAAAAAGTACCTGTCTTTCGGCTCTGTG |
| ProgR-3UTR-R1 (*NotI*) | ATCgcggccgcATGAAAACAACTAGCACTCTCCGTTCTGGTTTTCTCA |
| ProgR-5KOV-F1 | ACGCAAAGAATGGGTAAAGGTCTACAGCAGTGC |
| DHFR-5KOV-R1 | GGGAAAGTCACGCATATGGAACAGCACCGGG |
| DHFR-3KOV-F1 | CACTTTTTGGTGGGTCGGTTCTCCGTATTTGTATC |
| ProgR-3KOV-R1 | ACCCCTTCTACGCCGTGCTAGTCTATTGTCC |
| ProgR-ISP-F1 | AATGCGTTATCGTTCTGTATGCCGCTAGAG |
| ProgR-PD-3UTR-R1 | ATCcctaggACACTACACACCGCGAAACTCCTTTACGTTTAAACC |
| **Complementation of ΔTgMAPR** | |
| PtubProgR-comp-F1 *(ApaI)* | GACGGCGGCTGGAATGCAGCACC |
| PtubProgR-comp-HA-R1 *(HindIII)* | CGTTTTGGAGAGCGGGTCGTCGAGCATGTGCGGTGAGGATCCACACAGAAAGCTG |
| **Genomic insertion of TgCYP450mt-HA or TgMAPR-HA for generation of cKO** | |
| | **Insertion of C-terminal HA tag** |
| CYP-3HAgRNA.Mutag-R1 | cggcgattcacAACTTGACATCCCCATTTAC |
| CYP-3HAgRNA.Mutag-F1 | ttctgtgacaGTTTTAGAGCTAGAAATAGC |
| HACATRT-R1 | CGACGGCCAGTGAATTGTAATA |
| HACATRT-F1 | GGAAGTGGAGGACGGGAATTC |
| CYP-3HAV-R1 | GAATCTTCTGCCATCGACGGCCAGTGAAT |
| CYP-3HAV-F1 | GTTTCAAACCTCGGGCGGGAAGTGGAGGAC |
| ProgR-3HAgRNA.Mutag-R1 | gcggcaaaccAACTTGACATCCCCATTTAC |
| ProgR-3HAgRNA.Mutag-F1 | tgtcttcgcgGTTTTAGAGCTAGAAATAGC |
| ProgR-3HAV-R1 | CATGCATTTTGTCGAAAAAGGG |
| ProgR-3HAV-F1 | GCTTGCAGTAGGAAGTGGAGG |
| | **Insertion of inducible promotor** |
| CYP-PIgRNA.Mutag-R1 | ggctgccttgcAACTTGACATCCCCATTTAC |
| CYP-PIgRNA.Mutag-F1 | aacacaccaaGTTTTAGAGCTAGAAATAGC |
| PRHRT-R1 | GGTTGAAGACAGACGAAAGCAG |
| PRHRT-F1 | GAACTACGTGGACATTAAGTTCCATTC |
| CYP-PIV-R1 | CGATACTGAGAACTCGCAAC |
| CYP-PIV-F1 | GCATTTACAACTGTACGGCG |

**S1 Table. Cloning primers used for the study of TgMAPR and TgCYP450mt**
