# Supplementary material for: Function and regulation of a steroidogenic CYP450 enzyme in the mitochondrion of Toxoplasma gondii
Source: PLoS Pathog. 2023 Aug 31;19(8):e1011566. doi: 10.1371/journal.ppat.1011566 (PMC10499268; doi:10.1371/journal.ppat.1011566)
Supplement: S1 Data — (PDF) [file ppat.1011566.s012.pdf]

Fig 3A: Plaque area

Parental	DeltaTgMAPR
870	112
895	160
889	132
919	98
725	78
850	87
891	159
689	80
971	120
802	125
697	99
727	114
901	136
857	125
896	105
766	113
957	122
805	130
987	99
870	98
849	129
797	118
915	128
844	100
840	123
880	142
850	109
894	123
772	92
799	104
785	97
850	173
970	113
825	89
916	152
711	

Fig. 3B: Parasite counting

Parental (invaded)	Parental (attached)
27	2
30	3
29	1
26	3
31	2
25	1
35	4
30	2
26	3
27	2
24	4
28	3
26	2
24	2
25	2
27	4
26	2
28	3
24	1
27	2
31	2
30	2
	3
	1
	3
	2
	1
	3

Fig. 3D: cpm values

DeltaTgMAPR (invaded)	DeltaTgMAPR (attached)	Parental (Exp #1)
13	11	2541
12	10	2499
12	11	2678
12	9	
10	12	
10	9	
10	13	
12	10	
11	14	
13	10	
13	9	
12	9	
14	8	
14	8	
11	9	
12	9	
12	10	
13	11	
10	12	
14	13	
11	8	
13	9	
16	14	
13	9	
12	11	
14	12	
15	14	
10	13	
11	9	
12	8	
11	8	
15	10	
14	10	
14	11	
13	10	
	9	
	9	

DeltaTgMAPR (Exp #1)	Parental (Exp #2)	DeltaTgMAPR (Exp #2)	Parental (Exp #3)
1960	3312	2375	2104
1742	3601	2154	2255
2108	3879	2635	1998

DeltaTgMAPR (Exp #3)
1799
1820
1489

Fig. 5A: plaque area

Parental	Complemented	DeltaTgMAPR>7wks	DeltaTgMAPRad
774	810	98	399
641	775	111	613
701	699	125	456
599	710	115	580
556	874	112	722
751	777	107	470
655	901	124	664
589	756	99	552
658	845	137	637
602	688	132	424
651	638	122	701
702		133	533
666		116	532
645		102	655
684			501
670			499
			632
			751

Fig. 5B: parasite counting

Parental (invaded)	Parental (attached)	DeltaTgMAPRad (invaded)
31	8	24
32	6	26
29	7	25
32	9	19
39	8	27
28	9	27
27	7	17
26	8	23
31	8	24
31	7	23
29	9	24
31	6	19
33	7	26
30		26
32		
30		
31		
29		
33		
30		
28		
30		
29		
29		
32		
31		
31		
30		
32		
33		

Fig. 5C: cpm values

DeltaTgMAPRad (attached)	Parental (Exp #1)	Complemented (Exp #1)
10	4754	5406
12	5048	5812
9	4824	5701
8		
10		
10		
8		
9		
9		
12		
7		

DeltaTgMAPR<7wks (Exp #1)	DeltaTgMAPRad (Exp #1)	Parental (Exp #2)
3587	6102	5241
3698	5947	5147
3378	5784	5573

Complemented (Exp #2)	DeltaTgMAPR<7wks (Exp #2)	DeltaTgMAPRad (Exp #2)
5678	3883	7070
5410	3701	6874
5901	3978	7251

Parental (Exp #3)	Complemented (Exp #3)	DeltaTgMAPR<7wks (Exp #3)
3469	4246	2661
3099	4351	2514
3785	4155	2787

DeltaTgMAPRad (Exp #3)
4214
4424
4683

Fig. 1C: Plaque #

parental+ATc	iDeltaTgCYP450+ATc	iDeltaTgCYP450
38	40	26
24	30	36
22	23	37

Fig. 1D: Plaque area

parental+ATc
22.4
24.3
17.6

iDeltaTgCYP450+ATc	iDeltaTgCYP450
1.1	24.3
1.2	26.4
1.1	19.4

Fig. S6B: Relative amount

WT ergosterol	WT lanosterol	WT di-trienol	Parental ergosterol	Parental lanosterol
176	27	10	144	27
189	18	7	168	22
149	39	12	161	19

Parental di-trienol	SpDap1KO ergosterol	SpDap1KO lanosterol	SpDap1KO di-trienol
6	44	76	24
7	69	66	20
6	33	49	15

SpDap1KO+Dap1 ergosterol	SpDap1KO+Dap1 lanosterol	SpDap1KO+Dap1 di-trienol
149	78	15
132	61	17
164	59	8

SpDap1KO+TgMAPR ergosterol	SpDap1KO+TgMAPR lanosterol	SpDap1KO+TgMAPR di-trienol
123	33	7
144	41	3
139	31	5

SpDap1KO+vector ergosterol	SpDap1KO+vector lanosterol	SpDap1KO+vector di-trienol
81	37	8
70	42	8
68	32	10
