# Supplementary material for: Function and regulation of a steroidogenic CYP450 enzyme in the mitochondrion of Toxoplasma gondii
Source: PLoS Pathog. 2023 Aug 31;19(8):e1011566. doi: 10.1371/journal.ppat.1011566 (PMC10499268; doi:10.1371/journal.ppat.1011566)
Supplement: Attachment — Submitted filename: Rebuttal letter.docx [file ppat.1011566.s013.docx]

**Rebuttal letter**

Replies to the general comments of the Editors and Reviewers:

1) The organization of the manuscript and the presentation of data have been perceived as disordered: we have reconfigured the text and figures for better clarity, starting the revised manuscript by the last findings (Table 1 on steroid content in *Toxoplasma*), followed by the characterization of a cholesterol-metabolizing enzyme (TgCYP450mt -new Figure 1) and then on the characterization of a regulator of TgCYP450mt activity (TgMAPR from new Figure 2). The panels of each figure have also been reformatted for better style, spacing and color; statistical analyses and scale bars have been provided.
2) The execution and interpretation of experiments with AG-205 were considered as inaccurate: we decided to remove these data from the revised manuscript.
3) Although TgCYP450mt shares many characteristics (sequence features, activity, and localization) with mammalian enzymes involved in steroidogenesis, we have tempered our conclusions, opening the possibility that TgCYP450mt and TgMAPR may function in steroidogenesis and other pathways.

_____________________________________________________________________________________

**Answers to the major comments of Reviewer #1:**

*1- Concerning our assays on TgMAPR dimerization and membrane-association:*

Dimerization: In the revised version, we performed immunoblotting assays on freeze-thaw lysates of wild-type (WT) parasites using anti-TgMAPR antibody and observed TgMAPR expressed as a monomer and dimer (new Fig. 2A-P10L279). The initial assay showing TgMAPR dimerization performed on freeze-thaw lysates of *Toxoplasma* expressing TgMAPR-HA using anti-HA antibody, is confirmatory and has been moved to Fig. S5B.

Membrane-association: To confirm that TgMAPR behaves as an integral membrane protein, we performed a Western blotting analysis on WT parasites in which proteins were extracted in 1% Triton X-100 or 0.1 M Na_2_CO_3_. New Fig. 2B shows that TgMAPR partitions into the pellet phase of the Na_2_CO_3_ extract, similarly to the integral TgABCG protein and unlike the soluble GRA1, which partitions into the supernatant phase of the Na_2_CO_3_ extract (P10L284). A new Western blotting section has been added in Materials and Methods (P25L718).

*2- Concerning the drug target of the AG-205 compound:*

In the revised version, we deleted our data concerning the anti-*Toxoplasma* activities of AG-205 (ex. Fig. 3) as we concur that this study requires additional (future) investigations. We have identified AG-205 in a fluorescence image screen aiming to identifying new compounds targeting lipid pathways that have anti-proliferative properties against *Toxoplasma*. Interestingly, we found that AG-205 has an IC_50_ of 18 ± 3 µM for *Toxoplasma*, which is in the subtoxic concentration range for mammalian cells. We also found that AG-205-treated WT *Toxoplasma* but not AG-205-treated ΔTgMAPR display defects in the mitochondrion, but we agree that, at this stage, we do not have direct evidence that AG-205 targets TgMAPR.

*3- Concerning mouse infection assays:*

We specified that infected mice were monitored daily to report mouse survival versus death. The humane endpoints to terminate the experiments was euthanasia using carbon dioxide inhalation according to our animal protocol (P22L654).

**Answers to the minor comments of Reviewer #1:**

• *The title is hard to digest:* we provided a new title “Function and regulation of a steroidogenic CYP450 enzyme in the mitochondrion of *Toxoplasma gondii*”.

• Abstract and author summary: as suggested, we used the term “conditional knockdown”.

• *The introduction is fairly long with unnecessary information for the study*: we provided more concise information related to the topic of the manuscript and clarified that our goal was to extend our longstanding studies on cholesterol in *Toxoplasma* to metabolites derived from cholesterol.

• We used gene IDs from the two background strains ME49 and GT1 indifferently as these strains have the same ID number (315770 for CYP450mt and 276990 for MAPR) for the same gene, with sequences 100% identical.

• *Concerning the format and content of the figures to be improved*: we have taken into consideration all the suggestions of this reviewer: better alignment of panel letters and use of i, ii, iii for subpanels; clearer presentation of the images (larger, well-aligned, homogenous); separate channels at equal intensity for IFA; addition of scale bars and necessary controls that were missing. We also added the figure legend after each figure to facilitate the reading.

• *Concerning the format and imprecisions of the text*:

We added the line numbers in the margins of the manuscript.

• Results: *concerning our statement that an “ancestral” MAPR gene kept in the Sarcocystidae family*: We used the terminology of ‘ancestral’ as this adjective has been used in articles on phylogenic studies about the eukaryotic origin of MAPR family proteins (PMID: 28104494; PMID: 17583495; PMID: 28250339, PMID: 32278076). The authors refer to an ancestral MAPR protein that shares some sequence features with mammalian MAPR (such as a C-terminal cytochrome *b5* family heme/steroid binding domain) but lacks protein interaction motifs present in higher eukaryotic PGRMC proteins and proposed to be acquired later during evolution. Canonical PGRMC proteins have evolved motifs associated with sophisticated systems of PGRMC1-modulated regulation in multicellular organisms that are absent in TgMAPR. In this case, we would like to keep the appellation ‘ancestral’ for TgMAPR but we have replaced ‘kept’ by ‘possess’ in the sentence P9L250.

• “Displaying various subcellular localizations, MAPR proteins fulfill a broad range of functions related to cell survival, including cholesterol synthesis and homeostasis, steroid hormone production and signaling, and resistance to DNA damage-induced stress.” As suggested, we moved this information in the Introduction (P5L130) and summarized it in the Results section on MAPR sequence analysis as a reminder of the multifunctionality of MAPR proteins.

• *The in-gel heme assay is insufficiently described*: we have added an explanation of this assay in Materials and Methods (P25L741). For additional information, an in-gel heme assay is similar to the assay for immunodetection of antigens immobilized on filters using secondary antibody conjugated to HRP which catalyzes the oxidation of the luminol substrate, thus the in-gel heme assay takes advantage of the intrinsic peroxidase activity of the heme group of a denaturated protein and utilizes the same detection reagents of the enhanced chemiluminescence (ECL) Western blotting detection system. Briefly, protein suspensions are incubated for 15 min at 42^0^C in 124 mM Tris (pH 7), 20% glycerol, 4.6% SDS (no β-mercaptoethanol, no boiling). After electrophoresis on SDS-PAGE and transfer to nitrocellulose filters, the ECL detection reagents are used according to the manufacturer’s recommendations, with the detection solution added directly to the blot for 1 to 10 min. Under the denaturing electrophoretic conditions, only proteins containing covalently bound prosthetic group heme retain their heme and will be identified on blots by virtue of their heme peroxidase activity. First established on c-type cytochrome, this method of detection is applicable to any hemoprotein that retains its peroxidase activity.

• *Concerning the assay of HeLa cells expressing TgMAPR-HA*: This assay reveals that TgMAPR is expressed in the mitochondria in VERO cells, and was conducted (by genuine curiosity) because only cancerous cells express PGRMC1 in mitochondria, sharing thus with *Toxoplasma* this unique localization for a MAPR homolog. We moved this data in Figure S5A.

• *Concerning the localization of TgMAPR-HA in IMM/OMM:* we initially performed an immunoEM (IEM) staining on WT parasites using the anti-TgMAPR antibody but this antibody did not work well in our IEM protocol and gave a high, non-specific background in the host cell. We then performed IEM staining in TgMAPR-HA-expressing *Toxoplasma* using anti-HA antibody. We show that gold particles localized on the two mitochondrial membranes. There is no information about the fine localization of PGRMC1 in the mitochondria (i.e., on which membranes) of cancerous cells. We believe that the final destination of TgMAPR/PGMRC1 is the IMM (or at OMM-IMM junctions) where the CYP450 enzymes are located. Would it be the case, we then expect to also observe gold particles on the OMM, capturing TgMAPR crossing this membrane. We agree that we cannot rule out the possibility that the localization of TgMAPR to the OMM and IMM results from TgMAPR-HA overexpression. However, this strain behaves like WT parasites, with TgMAPR-HA expression as monomer/dimers, association with the mitochondrion and colocalization with the signal of anti-TgMAPR antibody. However, we tempered our findings on TgMAPR-HA in TgMAPR-HA-expressing parasites and moved these data to Figure S5B-D.

• *Concerning PCR gels in Fig. S3C (ex. Fig. S3A):* we agree that we should have designed primers in UTRs to show bands in the WT samples at different sizes than in the KO; we were confident that the *mapr* gene was deleted and restored in the complemented strain.

• *Concerning the different colors in the cell cycle graphs:* In the FlowJo software, the different colors allow the visualization of the percentage/density of a cell population in a specific phase of the cell cycle. The pseudo-color plot graphs for the cell cycle of WT *Toxoplasma* show high populations of parasites at 1N DNA phase (=light green peak) and at 2N DNA phase (=light blue peak), and very few parasites transitioning between 1N and 2N (=khaki peak). In contrast, the khaki peak is prominent in ΔTgMAPR^<7wks^, indicating severely dysregulated cell cycle. We provided this explanation in the Figure 4 legend (P39L1260).

• *Concerning the organelle shown in Fig. 4 (ex-Fig. 7A), panel ii*: this organelle is not an artifact of EM processing. This is the plant-like vacuolar compartment (PLVAC; PMID: 3621800) of *Toxoplasma*, acting like an endo-lysosomal compartment, with limited digestive functions but many transport activities. In extracellular parasites, PLVAC is a single, enlarged compartment, apically located, with indeed various unique shapes, e.g., squarish, polygonal or star-like. In replicating intracellular *Toxoplasma*, the PLVAC undergoes fragmentation into smaller vesicles that distribute throughout the parasite cytoplasm. The oddity is that in intracellular ΔTgMAPR^ad^ parasites, PLVAC persists as a single organelle as in extracellular WT parasites and is also aberrantly located at the basal end, suggesting an abnormal remodeling PLVAC following invasion. In the revised version, we provided a better explanation of this abnormal phenotype P16L477.

*• Concerning the localization of TgMAPR:* We provide additional evidence using IFA about the mitochondrial localization of TgMAPR and TgCYP450mt using anti-mitochondrial HSP70 antibody (more stringent than Mito-Tracker) in new Fig. 1B and new Fig. S5E, with PCC values > 0.5. When the signals of TgMAPR and TgCYP450mt were compared, in less than 5% of parasites, the TgMAPR signal was seen outside the TgMAPR signal (Fig. 7). Similarly, when the signals of TgMAPR were compared with that of HSP70, in few parasites, the TgMAPR signal slightly extends beyond the HSP70 signal (Fig. S5F). We believe that TgMAPR localizes to the mitochondrion, but is perhaps more concentrated on some areas on the mitochondrion than TgCYP450mt or HSP70, giving thus a stronger signal by IFA. We do not think this could be an apicoplastic staining for TgMAPR as this organelle is apical in *Toxoplasma*. We provide an alternative image for the co-staining of α-MAPR and α-HA antibodies in TgMAPR-HA-expressing parasites in Fig. S5C (ex-Fig. 3B) with quantification.

• *Concerning the inclusion of another mitochondrial membrane protein as negative control in the PLA*: For the revision, we performed new PLA assays using anti-HSP70 antibody in new Fig. S9 (P17L513), showing no PLA signal upon co-addition of antibodies against HSP70 and TgCYP450mt or TgMAPR. This indicates no interaction of HSP70 and either TgCYP450mt or TgMAPR in the mitochondrion despite strong overlapping staining in the mitochondrion seen by IFA. Although PLA is used to study protein-protein interactions in situ, we deleted the sentence, ‘TgCYP450mt or TgMAPR possibly forming stable conjugates in the parasite mitochondrion’. Our future plan is to identify all the partners for TgMAPR in co-IP assays. We add the separate DAPI channel in Fig. 8A.

• *Concerning a decrease of different steroids in the TgMAPR KO and TgCYP450 iKD lines*: we did not include more controls as, due to the slowed growth of mutants and repeated purification cycles of parasite samples, these assays require numerous large flasks with infected cells to isolate sufficient amounts of parasites but we intend to pursue steroid analysis on additional mutants.

• Discussion. We agree that our model (ex. Fig. 12) was too hypothetical, not summarizing our findings. We removed this figure but discussed the potential role of other effectors involved in cholesterol transport to the mitochondrion for metabolization (P19L547).

• *Concerning the discussion on CYP450 evolution*: we have shortened this paragraph but would like to keep its main message as it strengthens our findings on TgCYP450mt being the first mitochondrial enzyme identified in a primitive eukaryote that produces steroids. The origin of cholesterol-metabolizing enzymes in the eukaryotic lineage is still a matter of debate in the current literature, and our data point to Sarcocystidae among Alveolates as one point of origin.

• Materials and methods. All primer sequences are in Table S1. We just kept the sequence of the protospacer in the Materials and Methods section.

_____________________________________________________________________________________

**Answers to the major comments of Reviewer #2:**

*Point # 1 concerning the membrane-association of TgMAPR and its sub-localization in the mitochondrion:*

> Membrane-association: To confirm that TgMAPR behaves as an integral membrane protein, we performed a Western blotting analysis on WT parasites in which proteins were extracted in 1% Triton X-100 or 0.1 M Na_2_CO_3_. New Fig. 2B shows that TgMAPR partitions into the pellet phase of the Na2CO3 extract, similarly to the integral TgABCG protein and unlike the soluble GRA1, which partitions into the supernatant phase of the Na_2_CO_3_ extract (P10L284). A new Western blotting section has been added in Materials and Methods (P25L718).

> Gold particles distribution for TgMAPR in the mitochondrion: We performed immunoEM staining in TgMAPR-HA-expressing *Toxoplasma* using anti-HA antibody, and show gold particles localized on the two mitochondrial membranes. There is no information about the fine localization of PGRMC1 in the mitochondria (i.e., on which membranes) of cancerous cells. We believe that the final destination of TgMAPR/PGMRC1 is the IMM (or at OMM-IMM junctions) where the CYP450 enzymes are located. Would it be the case, we then expect to also observe gold particles on the OMM, capturing TgMAPR crossing this membrane. In this case, we tempered our finding on TgMAPR-HA sub-localizations on mitochondrial membranes and moved them in Figure S5B-D.

*Points #2 and #3 concerning the drug target of the AG-205 compound:*

In the revised version, we deleted our data concerning the anti-*Toxoplasma* activities of AG-205 (ex. Fig. 3) as we concur that this study requires additional (future) investigations. We have identified AG-205 in a fluorescence image screen aiming to identifying new compounds targeting lipid pathways that have anti-proliferative properties against *Toxoplasma*. Interestingly, we found that AG-205 has an IC_50_ of 18 ± 3 µM for *Toxoplasma*, which is in the subtoxic concentration range for mammalian cells. We also found that AG-205-treated WT *Toxoplasma* but not AG-205-treated ΔTgMAPR display defects in the mitochondrion, but we agree that, at this stage, we do not have direct evidence that AG-205 targets TgMAPR. As suggested by this reviewer, it will be interesting to examine whether AG-205 treatment leads to changes in steroid abundance.

*Points #4*, *#5 and #6 concerning the quantification of ΔTgMAPR phenotypes:*

We provide a new Table 6C listing the ΔTgMAPR cytopathies, highlighting which ones are restored in the culture-adapted mutant, plus a more detailed description of each cytopathy in the text (P13L387 and P16L477).

*Point #7 concerning controls of PLA using mitochondrial proteins:*

For the revision, we performed new PLA assays using anti-HSP70 antibody in new Fig. S9 (P17L513), showing no PLA signal upon co-addition of antibodies against HSP70 and TgCYP450mt or TgMAPR. This indicates no interaction of HSP70 and either TgCYP450mt or TgMAPR in the mitochondrion despite strong overlapping staining in the mitochondrion seen by IFA. Our future plan is to identify all the partners for TgMAPR in co-IP assays.

**Answers to the minor comments of Reviewer #2:**

*1. Concerning the primary sequence of the Neospora MAPR homologue longer (contaminated by introns) than for other MAPR homologues from Sarcocystidae.* We thank this reviewer for noticing the mistakes in the *Neospora* *caninum* MAPR sequence at the C-terminus. In new Fig. S4A, we show the Sarcocystidae MAPR sequences (including the one of *N. caninum*) truncated at the C-terminus as we want to document that *N. caninum* MAPR has the conserved cytochrome b5 family heme/steroid binding domain.

2. In new Figure S4B, we provided an alignment of TgMAPR with yeast Dap1 and human PGRMC1, showing conserved functional residues among these 3 proteins.

3. *Concerning PCR gels in Fig. S3C (ex. Fig. S3A):* we agree that we should have designed primers in UTRs to show bands in the WT samples at different sizes than in the KO; we were confident that the mapr gene was deleted and parental restored in this gene. We have indicated the position of the ProgR-5UTR-F1 and ProgR-3UTR-R1 primers the diagram.

4-6. *Concerning membrane association of TgMAPR insufficiently examined*: Please see my reply in major point#1. TgMAPR has a predicted molecular weight of 26.2-kDa and our calculation of the molecular weight of the band on the immunoblot is 27-kDa for TgMAPR and 27.5-kDa for TgMAPR-HA. We do not an explanation for this slightly higher size on blot gels.

7. 12 & 18. We apologize for these errors and replace TgPGRMC1 (the initial name we gave for the protein) by TgMAPR throughout the text.

9. For Fig. S3A (ex-Fig. S3C), we specified in Materials and Methods and the Figure legend that the PCR reactions verified that 7 out of the 14 tested subclones (shown in the lanes) were positive for integration of CAT resistance cassette and the 3xHA tag, which was further confirmed by western blot analysis using anti-HA antibody.

10. *Concerning the localization of TgMAPR:* We provide additional evidence using IFA about the mitochondrial localization of TgMAPR and TgCYP450mt using anti-mitochondrial HSP70 antibody (more stringent than Mito-Tracker) in new Fig. 1B and new Fig. S5E, with PCC values > 0.5. When the signals of TgMAPR and TgCYP450mt were compared, in less than 5% of parasites, the TgMAPR signal was seen outside the TgMAPR signal (Fig. 7). Similarly, when the signals of TgMAPR were compared with that of HSP70, in few parasites, the TgMAPR signal slightly extends beyond the HSP70 signal (Fig. S5F). We believe that TgMAPR localizes to the mitochondrion, but is perhaps more concentrated on some areas on the mitochondrion than TgCYP450mt or HSP70, giving thus a stronger signal by IFA. We do not think this could be an apicoplastic staining for TgMAPR as this organelle is apical in *Toxoplasma*. We provide an alternative image for the co-staining of α-MAPR and α-HA antibodies in TgMAPR-HA-expressing parasites in Fig. S5C (ex-Fig. 3B) with quantification.

11& 19. For the immunoEM staining, we incubated the sections with the primary antibody anti-HA, then with Protein A coupled to gold particles (no secondary antibody which would increase the distance between the antigen and gold particles). We rectified our mistake in Materials and Methods (P32L966).

13. We rectified the wrong citation of Figure 5.

14. *Concerning the green and blue colors on the histogram for cell cycle:* In the FlowJo software, the different colors allow the visualization of the percentage/density of a cell population in a specific phase of the cell cycle. The pseudo-color plot graphs for the cell cycle of WT *Toxoplasma* show high populations of parasites at 1N DNA phase (=light green peak) and at 2N DNA phase (=light blue peak), and very few parasites transitioning between 1N and 2N (=khaki peak). In contrast, the khaki peak is prominent in ΔTgMAPR^<7wks^, indicating severely dysregulated cell cycle. We provided this explanation in the Figure 4 legend (P39L1260).

15. We added the missing references related to the functions of the various CYP450 proteins.

16. *Concerning the accumulation of cholesterol in parasite strains defective in downstream steroid metabolism in Figure 11*: This is very good point that we will consider in our future lipid analysis. Our lipidomics in this study were solely focused on steroids and not on sterols or cholesteryl esters as potential sources of cholesterol for the mitochondrion and, thus, used specific solvents for steroid analysis. Interestingly, we observed an accumulation of lipid droplets in the ΔTgMAPR parasites that may suggest an upstream perturbation in the cholesterol cycle and metabolization in the mutant.

17. *Concerning the origin of the heme biosynthetic pathway in Toxoplasma*: we thank this reviewer for pointing out the differences between this pathway in *Toxoplasma* versus in plants.

_________________________________________________________________________________

**Answers to the major comments of Reviewer #3:**

*Point #1 concerning the survival of eukaryotic cells in the absence of steroidogenesis on contrast to Toxoplasma gondii:*

We agree that steroidogenic cells survive in vitro upon dysregulation of steroid production (e.g., upon ACTH-stimulated corticosteroid secretion). However, at the organismal level as documented for worms, plants, insects, mammals, steroids exert essential functions. For example, congenital lipoid adrenal hyperplasia in humans is a very severe disorder of steroidogenesis, characterized by minimal or absent serum concentrations of all steroids. So far, no information exists about the importance of steroids for a single cell organism such as a protist. Unlike mammalian cells, TgCYP450 is the only CYP450 enzyme present in *Toxoplasma*, and is essential in vitro and to survive in mice. The mitochondrial localization of TgCYP450mt and sequence similarities with mammalian steroidogenic enzymes, points to an involvement of this unique protein in steroid metabolism. We discuss the advantages of steroids for the parasite, with the possibility that these lipids may greatly contribute to the development and virulence of *Toxoplasma*. At this stage, we agree that we cannot rule out the possibility that the essentiality ofTgCYP450mt is due to its potential involvement in various other biosynthetic pathways, and not solely in steroidogenesis. We made this statement clearer in the revised version.

Our next step is to identify the components of the cholesterol import pathway (transducoesome), including the StAR the homolog to confirm the parasite’s ability to import cholesterol into the mitochondrion (comment #1). We believe this pathway is of central importance as the growth defects of ΔTgMAPR and iΔTgCYP450mt cannot be rescued by culture medium with serum containing steroids (comment #2).

For this study, we did not generate heme-binding mutants for TgMAPR as it would mimic the gene deletion phenotype based on published studies reporting that heme-mediated PGRMC1 dimerization is required for interaction with cytochromes P450 (comment #3). In the future, we intend to study the steroid profiles of various mutants including the adapted strain of ΔTgMAPR whose fitness is largely restored (comment #4). Would the steroid levels still be reduced in ΔTgMAPR^ad^, it would suggest that steroids are dispensable in vitro but perhaps not in vivo as ΔTgMAPR^ad^ did not regain virulence.

Regarding pleotropic functions for TgMAPR: this is plausible as mammalian MAPRs are chaperoning different P450 proteins, not solely for cholesterol-metabolizing enzymes. Moreover, we show that genetic ablation of the *mapr* gene from *Toxoplasma* results in various cytopathies, pointing to TgMAPR involvement in a wide range of functions in the mitochondrion such as in heme metabolism. We made this statement clearer in the revised version. Of note, unlike *Toxoplasma*, mammalian cells express two PGRMC proteins with overlapping functions: cells depleted of PGRMC1 have reduced levels of labile heme in mitochondria, ER, nucleus and cytosol while depletion of PGRMC2 reduces labile heme in the mitochondria and nucleus. In mice, PGRMC1 deficiency in the liver reduces the activity of many cytochrome P450s, which impacts mouse physiology. PGRMC2 deficiency in adipose tissues engenders mitochondrial dysfunctions. It could be interesting to know if double Pgrmc1/Pgrmc2 KO mice would survive.

*Concerning the cyst-like structure appearance of ΔTgMAPR*:

Our observations (e.g., accumulation of amylopectin granules and lipid droplets) lead to the inference that the deletion of the *mapr* gene initially causes a major stress event, causing a retardation of growth and increase of nutrient stores for survival. TgMAPR then seems to sustain tachyzoite proliferation, which is analogous to cancer cells in which PGRMC1 conveys better growth and to non-cancer cells in which PGRMC1 promotes several cancer phenotypes. In ΔTgMAPR tachyzoites, the biogenesis of amylopectin granules and defects in mitochondrial cristae would suggest a shift to glycolysis, as observed in bradyzoites. However, our IFA using antibodies against proteins expressed in bradyzoites (e.g., BAG, CST1) did not give any staining (P14L410). Additionally, our RNAseq analysis of ΔTgMAPR parasites did not reveal upregulated bradyzoite transcripts. The positive signal for TRITC-lectin we observed on ΔTgMAPR PV (stronger than on WT PV) is not uncommon and sometimes observed for slow-growing mutants. This peculiarity reflects a thickening of the PVM associated with sugar accumulation, but without the incorporation of cyst wall proteins as confirmed by the absence of a signal for CST1, a protein involved in cyst wall integrity and sturdiness for the long-term persistence of bradyzoite forms. We did not generate a type II strain lacking the *mapr* gene but our anti-TgMAPR antibody gives a signal in the parasite mitochondrion in ME49 parasites similar to RH parasites. Indeed, it would be interesting to study the virulence of ΔTgMAPR in a ME49 background.

*Point #2 concerning the reduction of steroid levels in our mutants:*

For this assay, we have cultivated the parasites in fibroblasts (up to fifty 75-cm^2^ cell culture flasks to collect sufficient amounts of purified parasites) for 3 days or until the monolayer is lysed. We agree with this reviewer that in this protocol, we have made the wild assumption that steroid production is steady throughout the tachyzoite intracellular cycle. Indeed after 3 days of cultivation, a large fraction of wild-type parasites was egressing while our mutants were still inside PV. In addition, we cannot disregard that in ΔTgMAPR parasites, which exhibit defects in mitochondrial cristae, the lower production of steroids could be attributed to impaired mitochondrial functions, which in turn impact steroidogenesis. Nevertheless, the mitochondrial damages upon *mapr* deletion were reversible as the mutant recovers overtime at least in vitro, with mitochondrial ultrastructure returning to normal. We discussed these two excellent points more evident in the text (P19L537): data in Fig. 9 (ex-Fig. 11) must be cautiously interpreted and not be regarded as providing definitive proof of impaired steroidogenesis.

*Concerning the ablation of enzymes more downstream in steroidogenesis and the use of aromatase inhibitors*: we intend to pursue our study on mitochondrial activities associated with steroidogenesis as well as investigate the role of individual steroids in *Toxoplasma* physiology and host-parasite function. We thank this reviewer for suggesting the use of aromatase inhibitors on *Toxoplasma* to examine the impact of these compounds on steroid hormone synthesis.

**Answers to the minor comments of Reviewer #3:**

- In Fig. 2 (ex-Fig. 1), we provided a positive control (hemoglobin) in an in-gel heme assay to confirm functional heme-binding domain in TgMAPR (panel D) through peroxidase activity, and a negative control (purified His-TgABCG) as a white suspension, in contrast to purified His-TgMAPR that has the brown color of hemoproteins (Panel E). Based on this evidence, we did not generate a TgMAPR mutant for the heme-binding domain to demonstrate heme binding on TgMAPR.

- For each IFA image, we have indicted the name of the protein (not the tag) for better clarity.

- In the revised version, we deleted our data concerning the anti-*Toxoplasma* activities of AG-205 (ex. Fig. 3) as we concur that this study requires additional (future) investigations. We have identified AG-205 in a fluorescence image screen aiming to identifying new compounds targeting lipid pathways that have anti-proliferative properties against *Toxoplasma*. Interestingly, we found that AG-205 has an IC_50_ of 18 ± 3 µM for *Toxoplasma*, which is in the subtoxic concentration range for mammalian cells. We also found that AG-205-treated WT *Toxoplasma* but not AG-205-treated ΔTgMAPR display defects in the mitochondrion, but we agree that, at this stage, we do not have direct evidence that AG-205 targets TgMAPR.

- We would like to keep separate figures on the phenotypes of ΔTgMAPR and iΔTgCYP450mt as they differ in functions, with TgCYP450mt being a canonical cholesterol-metabolizing enzyme and TgMAPR a stabilizer of cytochrome P450s involved in steroidogenesis and perhaps other pathways.

- We provide an inset in Table I with the mitochondrial enzymes present in mammalian cells that are required for steroidogenesis and where TgCYP450mt may be functionality related (blue enzymes). The genome of *Toxoplasma* contains many hydrolases, aromatases, sulfatase, oxidases that may function in a steroidogenic pathway, but their localization/function are largely unknown to ascertain their involvement in steroid production/conversion.

*__________________________________________________________________________________________________________________________________________________________________________*
