# Supplementary material for: Function and regulation of a steroidogenic CYP450 enzyme in the mitochondrion of Toxoplasma gondii
Source: PLoS Pathog. 2023 Aug 31;19(8):e1011566. doi: 10.1371/journal.ppat.1011566 (PMC10499268; doi:10.1371/journal.ppat.1011566)
Supplement: Attachment — Submitted filename: 2d Rebuttal.docx [file ppat.1011566.s014.docx]

**Replies to the Editor comments:**

Concerning additional functions of TgCYP450mt and TgMAPR in other systems: TgCYP450mt shares mitochondrial localization and sequence motifs with cholesterol-metabolizing CYP450 enzymes, yet the nature of the substrate/s and enzymatic step/s in steroid synthesis in which TgCYP450mt is involved remains to be determined (see new Fig. S10). At this stage, we cannot predict biosynthetic pathways other than steroidogenesis in which TgCYP450mt could also be engaged in the mitochondrion. Nevertheless, we added a paragraph in the Discussion on TgCYP450mt as a potentially multifunctional enzyme, which would explain the lethality of *Toxoplasma* in the absence of *cyp450mt* (P21L625).

The situation is different for TgMAPR as it may chaperone several enzymes based on published works on mammalian PGRMCs or yeast Dap1 that regulate the activities of various CYP450 that function in stress alleviation. By analogy, we observed multiple cellular defects in ΔTgMAPR parasites, pointing to the possibility that TgMAPR is involved in a wide range of mitochondrial functions. We made this statement in the revised version (P21L617), suggesting for example that TgMAPR may function as a heme chaperone for the parasite ferrochelatase, as reported for human PGRMC proteins; mammalian cells depleted of PGRMC1 or PGRMC2 have reduced levels of labile heme in organelles and cytosol. Overall, we concede that steroid deficiencies observed in TgMAPR-deficient parasites (perhaps in TgCYP450-deficient parasites, too) could be consequential to the loss of *mapr* (or *cyp450mt*). An explicit statement about the limitations of our study has been added at the end of the Results section (P18L538).

The editorial changes recommended by reviewers 4 and 2 have been incorporated into the text. We also replied to the concerns of all reviewers. Regarding the study of cholesterol import into the mitochondrion, it is our intention to functionally characterize an interesting StAR protein candidate in *Toxoplasma* that shares homology with mammalian STARD3, which is involved in cholesterol translocation across membranes including mitochondrial membranes. We added this information in the Discussion (P19L559).

We have modified our graphs and histograms to avoid the use of red-green colors. For the microscopy images: at the beginning of our study, we used the colors of the fluorophores Alexa Fluor 488 (green) and 594 (red) but as some of these images were acquired a long time ago, we are currently not able to change the colors. For the sake of homogenization between panels in the figures, we kept the colors of the Alexa Fluor 488 and 594 fluorophores for our more recently acquired images. If requested, we can change the colors of our recent images. Nevertheless, we thank the editor for this warning and we have taken note of the fact that the prevalence of red/green color blindness is high and, in the future, we will save our microscopy images using a set of different pseudo-colors.

________________________________________________________________________________________

**Replies to reviewer's responses to questions from Reviewer #4:**

● Concerning the organization of the manuscript and the presentation of data: the initial submission was perceived as disordered, so in the revised version, we made efforts to reorganize the presentation of our findings. To us, it seemed more logical to first present the characterization of a cholesterol-metabolizing enzyme (TgCYP450mt) along with the detection of steroids in the parasite, followed by the characterization of a regulator of TgCYP450mt activity, TgMAPR. Moreover, this leaves the study open for our ongoing work on the identification of many potential TgMAPR interactors as reported for PCRMCs proteins. We had hoped that this reconfiguration of the data would have satisfied the reviewer, but if not, we will be pleased to modify again the flow of the manuscript, if requested.

● Concerning the ‘imbalance’ of the TgMAPR vs. TgCYP450mt data: one novelty of our data resides in the detection of steroids produced by a parasite mitochondrial CYP450 enzyme, expanding the list of cell types/organisms with steroidogenesis. Our study of TgMAPR, however, took an expected turn because of the reversion, over time, of the phenotype of parasites lacking the *mapr* gene. This intriguing finding needed to be clarified at every step of the intracellular lifecycle. Thus, we provided more data to document the phenotype of ΔTgMAPR parasites at the onset of cultivation, to after their adaptation in culture. In contrast, parasites lacking the *cyp450mt* gene were inviable, without any signs of recovery or adaptation in culture. The analysis of TgCYP450mt knock-down parasites went therefore straightforward, and we performed standard assays to investigate the ability of iΔTgCYP450mt to invade cells, replicate and kill mice. For our manuscript, we did not perform EM observations on iΔTgCYP450mt parasites but we intend to inspect their ultrastructure in detail. We believe that the steroid deficiencies of the TgCYP450mt down-expressed parasites are expected to impact parasite functions beyond the mitochondria, potentially altering the morphology of many organelles. As such, we expect that an in-depth analysis will be required, which would be the basis for another manuscript. In addition, our future plans will include further enzymatic characterizations of proteins involved in steroidogenesis in *Toxoplasma* (and in other Coccidia), the analysis of the immunoregulatory roles of parasite steroids, as well as the identification of other mitochondrial binding partners of TgMAPR.

● Concerning the figures: we apologize the missing scale bars (all have now been provided) and we used Arial font in all Figures. Our primary goal was to provide fluorescence and EM images at the highest resolution and quality required for data analysis and interpretation, which we hope to have reached satisfactorily. During the revision, we worked on harmonizing the figures in the main text and supplemental information (19 figures total) and the presentation of the panels in each figure to our best; however, if still unsatisfactory and specific guidance is given to us, we will redesign the figures.

________________________________________________________________________________________

**Replies to minor comments from Reviewer #2**

*1.Line 59. “severe growth [impairment] in cultured cells”*

We rectified this typographical error.

*2. Line 75. “viable [in] vitro”*

We rectified this typographical error.

3. Line 144. “[www.tox[o]db.org](http://www.tox[o]db.org)”. Also check line 181: .org not .com

We rectified these typographical errors.

*4. Line 196. “TgCYP450 shares high sequence identity with mitochondrial CYP450 homologs present in fellow members of the Sarcocystidae family” What is the basis for concluding the CYP450 homologs in other Sarcocystidae are mitochondrial? Is there experimental evidence for this? If so, should cite. If not, then reword.*

We agree with this statement and delete ‘mitochondrial’ in the absence of experimental evidence.

*5. Line 229 and Figure 1E. “Data showed that all mice infected with iΔTgCYP450 parasites pretreated with ATc remained alive at Day 22”. Is this correct? From the graph, 80% of mice in the +ATc condition remain alive.*

We thank the reviewer for pointing out this mistake. It is indeed 80%, not 100% of mice that remain alive upon ATc addition.

*6. Figure 1E and Line 231. It is unclear from the description in the results and methods exactly how these experiments were performed. Did the authors pre-treat the parasites with ATc (which is what the text seems to suggest)? Did they then maintain mice with ATc in the drinking water? This should be clarified in the methods. If they pre-treated mice with ATc, then they cannot conclude that “TgCYP450mt is essential for parasite … survival in mice”. Rather, this experiment is testing the viability of parasites following pre-treatment in ATc.*

We provide additional details of our experimental settings for virulence assays in the legend of Figure 1E legend and Material and Methods (P23L677). Specifically, mice were infected with ATc-treated iΔTgCYP450 and ATc was also added in the drinking water of mice.

*7. Line 243. “instead exerting their function at a nongenomic level”. The meaning of “nongenomic level” isn’t clear.*

We clarified that ‘by exerting their function at a nongenomic level’, MAPR have (rapid) cellular effects that do not involve changes in gene expression.

*8. Lines 260-265. The alignment of the TgMAPR protein to the human homolog in the C-terminal region is not well-supported (eg the Y180 and RK192-193 residues in TgMAPR do not align with the proposed equivalent residues in the human protein). Concluding that the phosphorylation sites are conserved (especially in the absence of evidence that these are phospohorylation sites) is not well supported by the alignment.*

We rephrased the description of the motifs in the TgMAPR sequence related to conserved phosphorylation for regulation. Indeed, Y180 phosphorylation needs to be experimentally examined before proposing an earlier evolutionary origin of PGRMC homologs prior to Eumetazoa.

*9. Figure 2B. Should include molecular mass markers on these western blots.*

We added the position of MWt markers on the western blots.

*10. Line 397. “the mitochondrion exhibiting large cristae (Fig. 4A, panel iv). The mitochondria in this image are quite small and the cristae don’t seem obvious. Can the authors supply an image with better resolution and/or an image with greater magnification?*

We provide an inset in Fig. 4A (panel iv) showing a representative image of an abnormal mitochondrion with disorganized cristae. We removed ‘large’ cristae from the previous description in the absence of morphometric analysis of the cristae size in the mutant vs. WT parasites.

*11. Line 471. Do the authors mean to refer to Fig. 4A here?*

Yes, we rectified this error.

*12. Line 597. “this suggests a contribution of TgMAPR to parasite adaptation to a checkpoint that controls G1 progression”. The meaning here isn’t clear. Progression into or through G1 phase? It is conceivable that parasites are defective in an element of mitosis or, as the earlier part of this sentence and some of the authors’ data suggest, daughter cell formation/cytokinesis.*

We agree that inferring a role for TgMAPR in checkpoint control is too speculative. The sentence is deleted.

_______________________________________________________________________________________

**Replies to minor comments from Reviewer #4:**

*1. Out of pure curiosity, how come the authors do not detect any sterols in the host cells? Aren’t they supposed to also contain sterols?*

Fibroblasts contain sterols, with cholesterol being the main one. However, fibroblasts cannot produce steroids from cholesterol as they lack a steroidogenic pathway. Nevertheless, fibroblasts are responsive to exogenous steroids produced by other cells in vivo. Fibroblasts cultured in vitro can also respond to steroids added to the medium (ex. cortisol and prednisolone to stimulate their proliferation, DNA synthesis and collagen production). In our assays, to avoid contamination of the *Toxoplasma* samples, we cultivated infected fibroblasts in a steroid-free serum prior to parasite purification, thus we do not expect to detect steroids in host fibroblasts.

*2) In the introduction, nothing is mentioned about cholesterol-derived metabolites in protozoans. Is it because not a lit is known? The authors could mention that and it will help with the novelty of their findings.*

To our knowledge, the only modification of cholesterol identified in protozoa so far is esterification, with storage of cholesteryl esters in lipid droplets. Only one study has reported that *Trypanosoma cruzi* can metabolize dehydroepiandrosterone to precursors of testosterone, such as androstenedione and androstenediol, and convert exogenous androstenedione to testosterone, 17β-estradiol and estrone. The authors of this study did not investigate any metabolic enzymes involved in steroid production or whether cholesterol (salvaged from the host) or ergosterol could be the precursor of steroids in *T. cruzi*.

*3) Line 132: why is it of interest to know that PGRMC1 is overexpressed in malignant cancer cells and localizes to the mitochondria? The authors could either remove this sentence or explain why does it have a link with Toxoplasma gondii.*

We wanted to propose a parallelism between malignant cancer cells and *Toxoplasma* parasites because both express PGRMC1 and TgMAPR, respectively, solely in the mitochondria (not in other organelles as found for PCRMCs in non-cancerous mammalian cells or Dap1 in yeast). It is known that malignant cancer cells are very sensitive to pharmacological inhibition of PGRMC1 e.g., by the compound AG-205 that specifically binds to PGMRC1: addition of AG-205 to cancer cells results in cell growth inhibition in vitro and loss in drug resistance. Our unpublished data show that AG-205 has also a toxoplasmostatic effect, with an IC_50_ of 18 ± 3 µM for *Toxoplasma*, which is in the subtoxic concentration range for non-cancer mammalian cells. As these data are still preliminary, we delete ‘of interest’ in the Introduction.

*4) Line 144: How many genes in Toxoplasma contain ligand-binding domains typifying nuclear hormone receptors or the sequence motifs (e.g., Zn-finger, C4-type steroid receptor? I think the authors should be more specific. This will contribute to the evolutionary aspect of this manuscript, as we would expect protozoan to have a reduced number of receptors compared to mammals.*

We thank the reviewer for this suggestion as it could be informative to provide a list of nuclear hormone receptors or steroid-related enzymes. The genome of *Toxoplasma* contains annotation for many Zn-finger containing proteins and enzymes (e.g., hydrolases, aromatases, sulfatase, oxidases) that may function in a steroidogenic pathway, but their localization/function are largely unknown, making it difficult to ascertain their involvement in steroid production/conversion. Nevertheless, we provide in the Introduction (P5L1440) the gene ID of strong candidates for 3-oxo-5-alpha-steroid 4-dehydrogenase (SRD5A2) that converts testosterone into 5-alpha-dihydrotestosterone and progesterone or corticosterones into their corresponding 5-alpha-3-oxosteroids [TGME49_272180, TGME49_285240 and TGME49_304480]. We also included the gene ID for additional cytochrome b5 family heme/steroid binding domain-containing proteins [TGME49_240770; TGME49_276110 (ER localization) and TGME49_313580 (nucleus localization)].

*5) Line 187: Sequence similarity searches are limited, and structural homology should also be implemented: the authors should use HHPRED (*[*https://toolkit.tuebingen.mpg.de/tools/hhpred*](https://toolkit.tuebingen.mpg.de/tools/hhpred)*) to check for structural homology. In fact, can see with HHPRED that the TgCYP450mt shares structural homology to cytochrome p450 4F22 but not to the other proteins mentioned in the text.*

Structural homology is definitively relevant to provide functional features of a novel protein. However, the cytochrome P450 4F22 is a glycosylated microsomal protein involved in acylceramide synthesis in the ER/Golgi while TgCYP450mt is mitochondrial.

*6) Line 223: I think the WB showing depletion of TgCYP450mt should be included in the main text, in figure 1. The authors could remove some of the panels of figure 1B and put them in the supplementary figures instead (since they all show colocalization of the protein with TOM40).*

If agreeable to this reviewer, we would prefer to compile into one Figure (Fig. S3) all of our genetic constructs along with the experimental evidence of successful gene insertion or deletion.

*7) Line 252: How was the predicted transmembrane domain information obtained? The Toxoplasma database does not predict any TM.*

Although the ToxoDB did not predict a TMHMM for TGME49_276990, we used <https://cctop.ttk.hu/> for the prediction of transmembrane helices and topology of proteins: one transmembrane domain has been predicted for TgMAPR (with 81% reliability) See <https://cctop.ttk.hu/job/results/f32899d944a14260997bea5e2142aeca>.

We added the CCTOP server name in the Results. To verify this prediction, we performed a Na_2_CO_3_ extraction from parasite lysates (Fig. 2B).

*8) Line 280: I would add that the antibody was generated in mice.*

This has been added.

*9) Line 304: The authors use the hyperLOPIT data to help them with the characterization of TgCYP450mt (line 185), but then do not do that with TgMAPR They should include it. It seems that the predicted localization of TgMAPR is mitochondrion – soluble, but the authors show otherwise.*

In ToxoDB, no hyperLOPIT data for TGME49_276990 had been reported (no section for *Protein targeting and Localization*) but, indeed, the original manuscript lists TGME49_276990 as a mitochondrial protein. We only challenge the ‘soluble’ property, based on our cell fractionation data shown in Fig. 2B.

*10) Line 312: I do not understand why the authors generated an TgMAPR-HA to confirm the localization of the protein. The localization of TgMAPR with the anti-TgMAPR antibody already shows that? Or I am missing something. To me, the only experiment that the authors should perform is an IFA with their antibody and anti-HSP70.*

We generated a *Toxoplasma* strain stably expressing TgMAPR-HA with the dual intention to ascertain the localization of TgMAPR in the mitochondrion and to use this strain in the future for co-immunopurification and identification of potential TgMAPR partners. Indeed, it is known that PGRMC proteins can act as chaperones for different CYP450 proteins. As confirmatory only, we had moved our data on TgMAPR-HA localization to supplemental information (Fig. S5).

*11) Line 331, comments for figure S6A: This is a very important and exciting experiment. However I would like to see a lane where the authors just use the parental yeast strain, and a loading control I think that is the bare minumus for a western blot. Additionally, did the authors try to localize the protein by IFA? Since DAP1 localizes to the ER, with would be interesting to see where TgMAPR localizes. Would it be going to the ER or to the mitochondria?*

In Fig. S6A, we added the western blots showing the expression of 3xHA-SpDAP1 in *SpDap1Δ* (complemented strain). We agree that the localization of TgMAPR in yeast would have been interesting based on the experimental evidence that TgMAPR can rescue the SpDAP1Δ phenotype, but we did not perform this assay at that time. Our intuition would be an ER localization for TgMAPR in yeast, based on findings that Dap1 activates two P450 enzymes required for ergosterol synthesis (Erg11/Cyp51) and (Erg5/Cyp61) that are both located to the ER [PMID: 17276356].

*12) Line 340, pathway in Figure S6B: The pathway is unnecessarily complicated. It is also small, clogged and hard to read. Please highlight the enzyme, and just add the precursor and final products along with the intermediates you mention in the text.*

We removed from Table I the inset with the schematic representation of steroid biosynthesis from cholesterol in Vertebrates. This set of data is now in new Fig. S10. Plus, we highlighted in blue the mitochondrial enzymes with functions compatible with TgCYP450mt.

*13) Line 373: the authors should be more precise when they mention “poor fitness of ΔTgMAPR parasites”. Since they did the experiments, I think they are refering to invasion and egress defects.*

We refer to ‘fitness’ as encompassing the defects of ΔTgMAPR parasites in invasion, replication and egress, as represented as poor growth in our plaque assays.

*14) Line 555: a quick search in the Toxoplasma database shows that there are three annotated StAR domain-containing proteins: tgme49_223150, 231000 and 236660. What made the authors choose the one they mention over the other two?*

The StAR domain mediates the transfer of various lipids between membranes, and indeed, the ToxoDB contains 3 genes coding for StAR domain-containing proteins (STARD). In comparison, mammalian cells express 15 members of the STARD family, with the two founding members being STARD1 and STARD3 (MLN64) that bind cholesterol and move this lipid across membranes. We preferentially mentioned TGME49_236660 in our Discussionb as it has the closest homology with STARD3, which transports cholesterol to the mitochondria and to the cell membrane [PMID:12070139; PMID:19965586]. TGME49_223150 has close homology with STARD5, which is a bile acid transporter that may also bind cholesterol for intracellular trafficking. TGME49_231000 has close homology with STARD10, which is a phosphatidylcholine and phosphatidylethanolamine transporter. We specified this information in the Discussion (P19L559).

________________________________________________________________________________________

**Replies to major concerns from Reviewer #3**

*Role of steroidogenesis (SG) in Toxoplasma gondii: Using TgMAPR KO and iKDs of TgCYP450mt that are deficient for growth and have decreased levels of several of the steroids measured, the authors argue that steroidogenesis is essential for Toxoplasma growth and proliferation. However, steroidogenesis is not required for the survival of a eukaryotic cell (most cells in the human body are for example non-steroidogenic); and it is possible that these proteins have functions independently of SG that contributes to the negative effects of their deletion.*

We truly understand the arguments of the reviewer. We removed all statements about SG and steroids being essential for *Toxoplasma proliferation*; we just want to convey the message that SG is an ancient pathway.

a) TgMAPR manipulation: in the plausible hypothesis that TgMAPR is a multifunctional protein, thus an activator of many CYP450 enzymes, its loss would indeed affect several metabolic pathways, not solely SG. Genetic ablation of mammalian PGRMCs/yeast Dap1 or pharmacological inhibition of PGRMC1 with AG-205 leads to decreased cell growth and lower resistance to drugs/stress factors. Similarly, ΔTgMAPR parasites have severe growth defects, although partially reversable overtime in the absence of external stressors. With this in mind, our data are not unexpected, and we have made clearer in the manuscript that SG regulation could be one of the functions of TgMAPR (P21L617).

b) TgCYP450mt manipulation: TgCYP450mt shares mitochondrial localization and sequence motifs with cholesterol-metabolizing CYP450 enzymes, yet the nature of the substrates and enzymatic step/s in steroid synthesis in which TgCYP450mt is involved remains to be determined (new Fig. S10). Our findings show that *Toxoplasma* lacking *Cyp450mt* are inviable and upon conditional knockdown expression, the mutant lost virulence. We agree that by comparison to (non)steroidogenic mammalian cells, these data may be surprising. Possible interpretations would be that: 1) TgCYP450mt is a monofunctional enzyme acting upstream of a pathway that produces essential metabolites; or 2) TgCYP450mt is a multifunctional enzyme operational in different pathways, with a broad impact on parasite physiology. If TgCYP450mt has multiple substrates, our data point to sterol/steroid as one of them, but it is difficult to predict, at this stage, the nature of additional substrates, as mentioned in the Discussion (P21L625).

c) Steroid deficiency: the physiological importance of steroids for *Toxoplasma* is also unknown. During the evolution of multicellular organisms, hormone steroids have acquired specialized functions (e.g., reproduction, inflammation, salt balance). It is possible that steroids for an ancient unicellular organism have primitive yet important roles in metabolism, defense mechanisms or survival. In this case, the physiology of a protozoan and a mammal related to steroid utilization may not be equivalent.

*1) TgMAPR is a receptor that in mammals is not essential for steroidogenesis — why did the authors investigate this protein and not STAR that is required to transport cholesterol into the mitochondria that is required for SG?*

We chose to leave any mention of StAR domain-containing proteins (STARD) to the Discussion (P19L559), after the presentation of our lipidomic data aiming at detecting steroids in *Toxoplasma*, instead of in the Introduction as we deemed it too premature. As we identified steroids in the parasite, we followed up by reporting in the Discussion that the ToxoDB contains annotation for genes encoding STARD homologs. Among them, TGME49_236660 harbors a canonical C-terminal StAR-related domain and could be an interesting candidate for possible function in cholesterol import into the mitochondria, based on sequence homology with mammalian STARD3. However, its investigation will be performed in the context of new study that will be centered on all STARDs in *Toxoplasma* (3 total with TGME49_223150 close to STARD5 and TGME49_231000 close to STARD10). We sincerely thank the reviewer for proposing to generate *Toxoplasma* STARD mutants to further support our data on SG.

*2) Have the authors supplemented cells infected with SG-mutants (TgMAPR KO and iKDs of TgCYP450mt) with the steroids for which they are deficient? Does this rescue growth and or development?*

ΔTgMAPR and iΔTgCYP450mt were cultivated in medium/FBS containing steroids or in medium/FBS that was steroid-free, and no growth differences were observed between the culture conditions. Rescue experiments by supplementing with specific steroids is a pertinent assay to link the weakness of our mutants with impaired SG. However, this will not be straightforward as it will first require examining whether WT parasites can access exogenous steroids added to the culture medium (e.g., uptake assays with individual radiolabeled steroids). If this is the case, then we will need to perform preliminary controls to assess the optimal concentration of a specific steroid that would avoid potential cytotoxic effects on the host cell (e.g., cell cycle dysregulation) or the parasite. Our next step is indeed to analyze the sources of steroids for *Toxoplasma* (i.e., biosynthesis vs. salvage). We appreciate the proposed experiments of the reviewer but they will require time and much optimization to provide solid conclusions.

*3) Have the authors expressed heme-binding mutants and or enzymatic mutants of TgMAPR KO and iKDs of TgCYP450mt, respectively? How does this affect growth? It is regrettable these experiments were not added, as they would enhance the publication, but they are not essential.*

We did not engineer heme-binding deficient parasites for TgMAPR or TgCYP450mt. We thank the reviewer for this suggestion that would have allowed us to verify whether TgMAPR dimerization involves heme binding. Our current study, however, is focusing on other aspects of the SG pathway.

*4) The authors report an ‘adapted TgMAPR KO. Does this parasite line have higher levels of the steroids deficient in the TgMAPR KO? This would be the expected result if steroids are essential for parasite proliferation.
Not addressing this point (similar to 1 + 2) by performing the suggested experiment remains a major weakness of the manuscript; this could be one way to address the importance of steroids (ie. if the adapted TgMAPRKO is still able to make steroids; however if it too is steroid sufficient that would argue against an important role for steroids in parasite proliferation).* *Given the small scale of the observed effects, to convincingly demonstrate steroidogenesis impairment by the knockouts the experiment requires more replicates to allow for statistical analysis (Fig. 9). In principle, a non-targeting ko control is required for this kind of experiment and the omission of such warrants a justification. Furthermore, it is unclear why the most abundant steroid detected from toxoplasma, 17-hydroxypregnenolone, was not used in the wt vs ko comparison. The authors are strongly urged to improve this data as it represents a cornerstone of their findings.*

We agree that analyzing the steroid content of the adapted strain of ΔTgMAPR parasites would have provided evidence that SG is important or not for the parasite. Due to technical challenges, it was experimentally unfeasible due to the high number of culture flasks required, to compare WT, pre-adapted ΔTgMAPR, adapted ΔTgMAPR and iΔTgCYP450mt parasites in sufficient amounts. In the future, we plan to compare WT, pre- and adapted ΔTgMAPR; in the absence of these data on steroid content, we removed from the text any statements claiming that SG is important for *Toxoplasma* proliferation. Our data in Fig. 9 are means of two independent parasite preparations (with technical replicates per experiment). For our assays, we performed targeted metabolomics, focusing on the detection of major steroid hormones for which we had comparative standards, not on steroid precursors (ex. pregnenolone derivatives, such as17-hydroxypregnenolone for cortisol synthesis).

*6) Effect of TgMAPR and TgCYP450mt on steroidogenesis: The authors show that parasites deficient for TgMAPR and TgCYP450mt have decreased levels of various steroids (Fig. 11). How was this experiment conducted? Given the dramatically, how can the authors be sure this is reflecting a difference in steroid synthesis rather than development-stage synthesis (i.e. more SG occurs at later stages in the lytic cycle). Furthermore, how can the authors be sure that their deficiency is directly affecting SG rather than indirectly affecting mitochondria? Can enzymes more downstream in SG be ablated? Can aromatase inhibitors be tested for their effects on Toxoplasma SG and growth?*

We concede that steroid deficiencies observed in TgMAPR-deficient parasites (perhaps in TgCYP450-deficient parasites, too) could be consequential to the loss of *mapr* (or *cyp450mt*). An explicit statement about the limitations of the study (i.e., variation possibly due to development-stage difference, mitochondria physiology) has been moved to the end of the Results section (P18L538). We detailed our experimental procedure in Materials and Methods but if more information is needed, we would be happy to provide it. Our intention is to pursue our study on the mitochondrial activities associated with SG, including identifying downstream enzymes and investigate the role of individual steroids in *Toxoplasma* physiology and host-parasite function. We thank the reviewer for suggesting the use of aromatase inhibitors on *Toxoplasma* to examine the impact of these compounds on steroid hormone synthesis.

________________________________________________________________________________________
